# Multi-Strategy Improved Pied Kingfisher Optimizer for Solving Constrained Optimization Problems

**DOI:** 10.3390/biomimetics11050335

**Published:** 2026-05-11

**Authors:** Hongmei Bai, Taosuo Wu, Jianfu Luo, Na Ta

**Affiliations:** 1 School of Mathematics and Physics, Hulunbuir University, Hailar 021008, China; baihongmei@hlbec.edu.cn; 2 School of Engineering, Hulunbuir University, Hailar 021008, China; wutaosuo@hlbec.edu.cn; 3 School of Artificial Intelligence and Big Data, Hulunbuir University, Hailar 021008, China; tanaa@hlbec.edu.cn

**Keywords:** pied kingfisher optimizer, multi-strategy improvements, constrained optimization problems, engineering optimization applications

## Abstract

This paper proposes a multi-strategy improved pied kingfisher optimizer (MSIPKO), a novel metaheuristic algorithm designed to address constrained optimization problems (COPs). COPs are widely encountered in engineering and industrial applications and are characterized by complex constraints that restrict the feasible solution space and often lead to multiple local optima. To enhance the performance of the original pied kingfisher optimizer (PKO), three strategies are incorporated: (i) a reverse differential crossover mechanism to improve global exploration and maintain population diversity; (ii) an enhanced diving-fishing operator to strengthen local exploitation; and (iii) an improved commensalism phase to enrich search directions and increase robustness. The performance of MSIPKO is evaluated on 12 benchmark functions from the IEEE Congress on Evolutionary Computation 2006 (CEC 2006) test suite and six classical engineering optimization problems. Experimental results demonstrate that MSIPKO outperforms several state-of-the-art algorithms in terms of optimization accuracy, convergence speed, and stability, particularly for high-dimensional, nonlinear, and multi-constrained problems. Moreover, MSIPKO achieves superior or comparable solutions with fewer function evaluations, indicating its high efficiency and adaptability. These results confirm that MSIPKO is a promising tool for solving complex real-world constrained optimization problems. Future work will focus on extending the proposed algorithm to multi-objective and large-scale optimization scenarios.

## 1. Introduction

Constrained optimization problems are a common challenge in the field of optimization. In real-world engineering scenarios, such as production planning and scheduling, most problems are subject to various constraints due to the scarcity of resources in the production process or technological limitations. Therefore, effectively solving such problems is of critical importance.

Compared to unconstrained optimization problems, constrained optimization problems are more complex due to the presence of constraints. When there are numerous constraints, the feasible solution space may become very limited. Additionally, if the feasible regions are disjoint, the problem may have multiple local optimal solutions. As a result, constrained optimization problems are significantly more difficult to solve than unconstrained ones. Research on the solution of these problems holds great theoretical significance and practical value.

### 1.1. Description of Constrained Optimization

Generally, a constrained optimization problem can be described by Formula (1). which is widely adopted in the literature and also used in our previous work [1].

(1) $$\begin{array}{ll} & \min\;\;f(x)\\ s.t.\;\; & g_{i}(x)\leq 0,i=1,2\ldots ,p\\ & h_{j}(x)=0,j=1,2,\ldots ,q\\ & u_{k}\leq x_{k}\leq v_{k},x\in R^{n},k=1,2,\ldots ,n \end{array}$$

where f(x) represents the objective function. $g_{i}(x)\leq 0$ is the *i*-th inequality constraint in optimization problem in Formula (1), and p is the number of inequality constraints. $h_{j}(x)=0$ is the *j*-th equation constraint, and q is the number of equation constraints. $u_{k}$ and $v_{k}$ are the upper and lower bounds of $x_{k}$, respectively. The set $D=\{x\in S|g_{i}(x)\leq 0,h_{j}(x)=0,$ i=1,2…,p,j=1,2,…,q} that meets all inequality and equality constraints in the search space $S=\{u_{k}\leq x_{k}\leq v_{k},$ $x\in R^{n},k=1,2,\ldots ,n\}$ is called the feasible region of the constrained optimization problem in Formula (1). If a group solution x∈D, *x* is called a feasible solution; otherwise, it is called an infeasible solution.

For (1), the distance from an individual *x* to the feasible region *D* is defined by (2).

(2) $$G(x)=\sum_{i=1}^{p}\max\left\{g_{i}(x),0\right\}+\sum_{j=1}^{q}\max\left\{\left|h_{j}(x)\right|-\delta ,0\right\}$$

Here, G(x) is called the violation function. $\max\left\{g_{i}(x),0\right\}$ (i=1,2,…,p) represents the violation degree of *x* in the *i*-th inequality constraint. $\left|h_{j}(x)\right|(i=1,2,\ldots ,q)$ represents the violation degree of *x* in the *j*-th equation constraint, where δ is the tolerance parameter of the equation constraint and δ=0.0001.

### 1.2. Related Work

When applying metaheuristic algorithms to constrained optimization problems (COPs), existing studies mainly involve the design of effective constraint-handling strategies, the development of high-performance optimization algorithms, and their applications in various engineering domains.

(1) Constraint-handling methods

From the perspective of constraint handling, classical approaches mainly include the penalty function method [2] and Deb’s rules [3]. The penalty function method incorporates constraint violations into the objective function, while Deb’s rules rank solutions based on feasibility and objective values. These methods are widely used due to their simplicity and ease of implementation. However, they are often sensitive to parameter settings and may suffer from slow convergence or premature stagnation when dealing with complex constrained problems.

To overcome these limitations, several advanced strategies have been proposed, such as transforming constraints into bi-objective formulations [4], the ε-constraint method [5], and dual-population strategies [6]. These approaches improve the balance between constraint satisfaction and objective optimization, thereby enhancing adaptability to complex constrained problems. Nevertheless, they usually introduce additional computational overhead and increase algorithmic complexity.

(2) Swarm intelligence algorithms and their improvements

From the perspective of algorithm design, a wide range of metaheuristic algorithms have been developed in recent years, particularly in the field of swarm intelligence optimization. In the past five years, more than 100 swarm-based optimization algorithms have been proposed, reflecting the rapidly growing research interest in this area [7,8]. Representative examples include Hawkfish Optimization Algorithm (HFOA) [9], Bezier Curve Optimization (BCO) [10], Dung Beetle Optimization (DBO) [11], Polar Lights Optimization (PLO) [12] and Sequoia-ecology-based Metaheuristic Optimization Algorithm (SMOA) [13]. Despite differences in inspiration and mathematical modeling, these algorithms share several common characteristics. Most of them adopt population-based search frameworks and utilize iterative update mechanisms to balance global exploration and local exploitation. In general, they exhibit strong exploration capability in the early search stages and can effectively avoid premature convergence to some extent.

However, these algorithms still face several inherent limitations when applied to constrained optimization problems. Complex constraints often lead to irregular, narrow, or even disconnected feasible regions, which significantly increase the difficulty of locating feasible and high-quality solutions. In such scenarios, many candidate solutions tend to fall into infeasible regions, making it difficult to maintain population diversity within the feasible space. In addition, the presence of multiple disjoint feasible regions may cause algorithms to converge prematurely to local feasible areas, thereby limiting global search capability. Furthermore, achieving an effective balance between exploration and exploitation under strict constraints remains challenging.

Therefore, developing metaheuristic algorithms with stronger adaptability, better diversity maintenance, and more effective search mechanisms for complex constrained spaces remains an important research direction.

To further enhance the performance of metaheuristic algorithms in constrained scenarios, many researchers have proposed improved or hybrid approaches by integrating multiple strategies. For example, Yu [14] proposed an enhanced Aquila Optimizer Algorithm (EAOA) incorporating restart strategies, opposition-based learning, and chaotic local search, and validated its effectiveness on several engineering optimization problems. Sun [15] developed a fuzzy logic-constrained particle swarm optimization algorithm (FILPSO-SCAε), which integrates a Spearman correlation-based adaptive ε constraint-handling method to dynamically balance constraint and objective information. In addition, fuzzy logic is employed to adaptively adjust key parameters, thereby improving search performance. Li [16] introduced an adaptive multi-objective transformation technique (AMaOTCO), which transforms constrained optimization problems into multi-objective ones by combining objective functions with weighted constraint violations, achieving improved convergence efficiency. Furio [17] proposed a hybrid grey wolf–JAYA algorithm (SHGWJA), which integrates elite strategies and adaptive perturbation mechanisms, and employs a penalty function to handle multi-constraint problems. The proposed method has been successfully applied to various engineering applications, such as structural optimization and path planning.

Although these approaches improve convergence behavior and solution quality to some extent, their performance gains often rely on complex hybrid strategies or multiple mechanisms. This may increase algorithmic complexity and reduce general applicability, especially in problems with highly complex constraint structures.

(3) Engineering applications

In practical applications, some studies have integrated algorithm improvements with constraint-handling strategies to address complex engineering optimization problems. These applications span a wide range of domains, including energy system scheduling [18,19,20] and optimal power flow in power systems [21,22]. In addition, various studies have explored more complex constrained scenarios.

For instance, Boualem [23] proposed an adaptive coordinate system-constrained differential evolution algorithm (ACS-CDE), which utilizes multiple coordinate systems and adaptive selection mechanisms to balance exploration and exploitation. Yang [24] developed an improved sandworm optimization algorithm (ISSA) for structural optimization problems, incorporating enhanced initialization and update strategies combined with penalty-based constraint handling. Abdollahzadeh [25] introduced a hybrid algorithm integrating the puma optimizer with neighborhood search for marine route optimization under multiple constraints. Similarly, Wang [26] proposed a multi-population adaptive optimizer (MACSGWO) for UAV path planning in complex three-dimensional environments. Other studies [27,28] have also applied metaheuristic algorithms to solve constrained optimization problems in similar engineering domains. In addition, Chen et al. [29] and Huang et al. [30] demonstrated the effectiveness of improved metaheuristic algorithms in industrial scheduling and portfolio optimization problems, respectively. These studies show that metaheuristic algorithms have achieved promising performance in various engineering applications with complex constraints.

However, despite these successes, most existing approaches are highly problem-dependent and often rely on carefully designed hybrid strategies or constraint-handling mechanisms. As a result, their generalization ability and robustness in handling diverse and highly complex constrained optimization problems remain limited.

(4) Summary of existing studies

Based on the above review, existing studies on constrained optimization problems can be broadly categorized into three main groups: constraint-handling strategies, swarm intelligence optimization algorithms, and their applications in engineering domains.

Constraint-handling strategies, including classical methods such as penalty functions and Deb’s rules, as well as advanced approaches like ε-constraint and multi-objective transformation techniques, provide effective mechanisms for handling feasibility conditions. However, their performance often depends heavily on parameter settings and may introduce additional computational complexity.

Swarm intelligence algorithms and their improved variants have demonstrated strong global exploration capabilities and have been widely applied in solving complex optimization problems. Nevertheless, these algorithms still face challenges in maintaining population diversity and achieving a proper balance between exploration and exploitation, especially in constrained search spaces.

In terms of engineering applications, metaheuristic algorithms have achieved promising results in various domains, such as energy systems, power systems, and structural optimization. However, many of these methods are highly problem-dependent and rely on carefully designed hybrid strategies, which may limit their generalization ability in diverse constrained optimization scenarios.

To further synthesize the above studies, a structured comparison of representative methods in terms of their advantages and limitations is provided in Table 1.

(5) Discussion and research gaps

Despite the significant progress achieved in constraint-handling strategies, swarm intelligence algorithms, and their engineering applications, several critical challenges remain in solving constrained optimization problems.

First, existing constraint-handling methods often struggle to effectively guide the search process in complex feasible spaces. In problems with irregular, narrow, or disconnected feasible regions, many algorithms tend to generate a large number of infeasible solutions, which reduces search efficiency and hinders convergence toward high-quality feasible solutions.

Second, most swarm intelligence algorithms still face difficulties in maintaining population diversity and achieving a proper balance between global exploration and local exploitation under strict constraint conditions. As a result, these algorithms are prone to premature convergence within local feasible regions, limiting their ability to explore multiple promising areas.

Third, although hybrid and improved algorithms can enhance performance by integrating multiple strategies, their effectiveness often relies on problem-specific designs and carefully tuned parameters. This reduces their robustness and generalization ability when applied to diverse and complex constrained optimization scenarios.

Therefore, there is a need to develop more effective optimization algorithms that can better handle complex constraint structures, maintain population diversity within feasible regions, and achieve a more balanced and robust search process. These challenges motivate the development of the proposed method in this study.

### 1.3. The Introduction of PKO

The pied kingfisher optimizer (PKO) is a novel swarm intelligence algorithm proposed by Bouaouda [31], inspired by the unique foraging behavior of the pied kingfisher, including hovering, diving, and its symbiotic relationship with Eurasian otters. The algorithm innovatively incorporates three phases: the hovering phase for global search, the diving phase for local exploitation, and the symbiotic phase for balancing exploration and exploitation. By mathematically modeling these behaviors into optimization strategies, PKO has been validated on 29 CEC-2017 benchmark functions and 10 CEC-2020 benchmark functions. Experimental results demonstrate that PKO outperforms many advanced algorithms in terms of solution quality, convergence speed, and avoiding local optima. Despite its superior performance in complex search spaces, PKO has certain limitations. For instance, the algorithm lacks a mutation mechanism, leaving room for improvement in its global search capabilities. Additionally, during the symbiotic phase, the algorithm occasionally chooses to keep the population stationary, which suggests potential enhancements to its search performance in this stage.

However, several limitations have been observed in the original PKO. First, it lacks any explicit mutation or perturbation mechanism throughout its iterative process, which limits the algorithm’s ability to escape local optima. As a result, the population tends to converge prematurely, especially when navigating complex, multimodal, or high-dimensional search spaces where diverse exploratory behavior is crucial. Second, the hovering and diving phases adopt a centralized update strategy that relies heavily on elite or globally best individuals. While this may accelerate convergence in simple landscapes, it significantly reduces the diversity of the population and restricts the algorithm’s capacity to explore less-exploited regions of the search space. In scenarios involving rugged fitness landscapes or disjoint feasible regions, this behavior can severely compromise the global search capability. Third, the symbiotic phase lacks directional learning mechanisms guided by fitness differences or relative advantage among individuals. The update rule often leads individuals to randomly drift toward other peers without considering solution quality, resulting in inefficient local exploitation and slow convergence in the later stages of the search. This inefficiency becomes more pronounced in fine-tuning phases where precision and gradient-like adjustment are essential.

### 1.4. The Main Contributions and Contents of This Paper

To address the limitations of existing methods in solving complex constrained optimization problems, this paper proposes a multi-strategy improved pied kingfisher optimizer (MSIPKO). The main contributions of this study are summarized as follows:

(1) A reverse differential crossover mechanism is proposed by integrating reverse learning with differential mutation, which effectively enhances global exploration capability and maintains population diversity, especially in complex feasible regions.

(2) An enhanced diving-fishing operator is designed to strengthen local exploitation performance, thereby improving convergence accuracy and accelerating the search process.

(3) An improved commensalism phase with a novel guidance mechanism is introduced to enrich search directions, leading to improved solution quality and robustness of the algorithm.

(4) Extensive experiments on IEEE Congress on Evolutionary Computation 2006 (CEC 2006) benchmark functions and classical engineering optimization problems demonstrate that the proposed MSIPKO achieves competitive or superior performance in terms of optimization accuracy, convergence speed, and stability compared with several state-of-the-art algorithms.

These contributions distinguish MSIPKO from existing hybrid metaheuristic approaches and provide an effective solution for complex constrained optimization problems.

The remainder of this paper is organized as follows. Section 2 introduces the basic pied kingfisher optimizer (PKO). Section 3 presents the proposed MSIPKO in detail. Section 4 reports the experimental results, including benchmark functions and engineering optimization problems. Finally, Section 5 concludes the paper and discusses future research directions.

## 2. The Process of PKO

The proposal of this algorithm is inspired by the living habits of pied kingfisher, considering their behaviors such as perching, hovering, diving, and symbiosis. The specific process is shown below. The mathematical formulations presented in this section follow the original PKO algorithm proposed in [31].

### 2.1. Initialization

Similar to many swarm intelligence optimization algorithms, the PKO algorithm initiates the search process by randomly generating a set of initial solutions in the search space as the first attempt. The initial population generation is shown in Formula (3).

(3) $$\begin{array}{l} X_{i,j}=LB+rand\times \left(UB-LB\right)\\ i=1,2,\ldots ,N\;\;\;\;\;\;\;\;\;\;\;j=1,2,\ldots ,\dim \end{array}$$

where $X_{i,j}$ means the position of the *i*-th individual in the *j*-th dimension. *rand* is a random number within the interval [0, 1]. *LB* and *UB* respectively represent the lower and upper bounds of the search range.

### 2.2. Perching and Hovering Strategies (Exploration Phase)

The exploration phase of the PKO algorithm was inspired by the habitat and hovering behavior of pied kingfisher. Based on observations of pied kingfisher in their natural habitats, they alternate between habitat attacks and hovering attacks according to different factors. The location of the individual being searched is updated based on the foraging activities of the pied kingfisher, as shown in Formula (4).

(4) $$\begin{array}{l} X_{i}\left(t+1\right)=X_{i}\left(t\right)+\left\{\begin{array}{l} \alpha \cdot T_{1}\cdot \left[X_{k}\left(t\right)-X_{i}\left(t\right)\right],rand\geq 0.5\\ \alpha \cdot T_{2}\cdot \left[X_{k}\left(t\right)-X_{i}\left(t\right)\right],rand<0.5 \end{array}\right.\\ i,k=1,2,\ldots ,N\;\;\;\;\;\;\;\;\;\;\;\;i\neq k \end{array}$$

where $X_{i}\left(t\right)$ and $X_{i}\left(t+1\right)$ respectively represent the solution vector of the *i*-th individual in iteration t and t+1. $X_{k}\left(t\right)$ means the solution vector of the *k*-th individual in iteration t. The population size is *N*.

The calculation method for parameter α is shown in Formula (5).

(5) $$\alpha =2\times randn\left(1,\dim\right)-1$$

where *randn* means random numbers between 0 and 1 that follow a normal distribution. dim is the length of the solution vector $X_{i}$, which is the dimension of the objective function.

The parameter *T* represents the strategy selection of perching or hovering, and the calculation method is shown in Formulas (6)–(9).

#### 2.2.1. Perching

In their habitat strategy, pied kingfishers typically inhabit objects such as trees, rocks, or electrical wires to search for prey. The calculation formula is shown in Formulas (6) and (7).

(6) $$T_{1}=\left[\exp\left(1\right)-\exp{\left(\frac{t-1}{Max\text{\_}Iter}\right)}^{\frac{1}{BF}}\right]\cdot \cos\left(crest\text{\_}angles\right)$$

(7) crest_angles=2π×rand

where Max_Iter is the number of maximum iterations. *BF* stands for beating factor, which is set to 8 in this algorithm. The crest angle of a pied kingfisher enhances its field of view for detecting prey from a distance and aids in focusing on prey during hunting.

#### 2.2.2. Hovering

In the hovering strategy, the pied kingfisher maintains its position in the air by rapidly flapping its wings, and its mathematical model is shown in Formulas (8) and (9).

(8) $$T_{2}=BeatingRate\cdot {\left(\frac{t}{Max\text{\_}Iter}\right)}^{\frac{1}{BF}}$$

(9) $$BeatingRate=rand\cdot \frac{PKO\text{\_}Fitness\left(j\right)}{PKO\text{\_}Fitness\left(i\right)}$$

where the *i*-th and *j*-th individual’s fitness are respectively $PKO\text{\_}Fitness\left(i\right)$ and $PKO\text{\_}Fitness\left(j\right)$.

### 2.3. Solid Samples Desorption and Sustainability

Pied kingfishers are known for their diving and hunting behavior, which was used for local searches during the development phase. The calculation formula is shown in Formulas (10)–(13).

(10) $$X_{i}\left(t+1\right)=X_{i}\left(t\right)+HA\cdot ο\cdot \alpha \cdot \left[b-X_{best}\left(t\right)\right],\;\;\;\;\;i=1,2,\ldots ,N\;\;\;\;\;\;\;$$

(11) $$HA=rand\cdot \frac{PKO\text{\_}Fitness\left(i\right)}{Best\text{\_}Fitness}$$

(12) $$ο=\exp{\left(\frac{-t}{Max\text{\_}Iter}\right)}^{2}$$

(13) $$b=X_{i}\left(t\right)+{ο}^{2}\cdot randn\cdot X_{best}\left(t\right)$$

where HA and ο are the parameters related to hunting ability.

### 2.4. Commensalism Phase (Local Escape Phase)

The symbiotic relationship between pied kingfisher and otters was used to simulate the escape phase, and the calculation formula is shown in Formulas (14) and (15).

(14) $$X_{i}\left(t+1\right)=\left\{\begin{array}{l} X_{m}\left(t\right)+ο\cdot \alpha \cdot \left|X_{i}\left(t\right)-X_{n}\left(t\right)\right|,rand>1-PE\\ X_{i}\left(t\right),\;\;\;\;\;\;\;\;\;\;\;\;\;\;\;\;\;\;\;\;\;\;\;\;\;\;\;\;\;\;\;\;\;\;\;\;\;\;rand\leq 1-PE \end{array}\right.$$

(15) $$PE=PE_{\max}-\frac{\left(PE_{\max}-PE_{\min}\right)\cdot t}{Max\text{\_}Iter}$$

where $X_{m}\left(t\right)$ and $X_{n}\left(t\right)$ respectively mean two random individuals of iteration *t*. *PE* represents predatory efficiency of the pied kingfisher. $PE_{\max}=0.5$, $PE_{\min}=0$.

### 2.5. The Flow of PKO

This section presents PKO’s framework in pseudocode form, as shown in Algorithm 1. The pseudocode outlines the three main phases of PKO—hovering exploration, diving exploitation, and commensalism cooperation—and details how these phases are executed within the iterative optimization process.
**Algorithm 1: Pseudocode of PKO****Input:** Population total number *N*; the number of optimization iterations *MaxIt*. **Output:** The optimal solution of PKO (Best_fitness), optimal solution vector *X*_best_.**1****Initialize** the PKO population positions according to Section 2.1.**2**Calculate the pied kingfisher fitness values**3****while** (*t* < *MaxIt* + 1) **do****4**  **for** i = 1:*N* **do****5**       **if** (rand < 0.8) **then****6**      % Exploration phase**7**      **if** (rand > 0.5) **then****8**        Compute *T*_1_ according to Formulas (6) and (7)**9**        Update the position of pied kingfisher using Formula (4)**10**      **else****11**        Compute *T*_2_ according to Formulas (8) and (9)**12**        Update the position of pied kingfisher using Formula (4)**13**    **end****14**    **else****15**      % Exploitation phase**16**        Update the position of pied kingfisher using Formula (10)**17**    **end****18**    If the newly generated solutions are superior to the previous ones, then replace them. Set best position as the location of best fitness.**19**    **if** (rand > (1 − *PE*)) **then****20**       Update the position of pied kingfisher using Formulas (14) and (15)**21**    **else****22**       Update the position of pied kingfisher using Formulas (14) and (15)**23**    **end****24**    Calculate the fitness values of pied kingfisher**25**    If the newly generated solutions are superior to the previous ones, then replace them. Set best position as the location of best fitness.**26**    *t* = *t* + 1**27**  **end for****28****end while****29**Return Best_fitness and *X*_best_.

## 3. Materials and Methodology

### 3.1. Overview of the Proposed MSIPKO Algorithm

The original PKO algorithm has demonstrated promising performance in solving optimization problems. However, similar to many population-based metaheuristic methods, it still suffers from several limitations, such as insufficient exploration ability in early iterations, lack of effective mutation mechanisms, and potential premature convergence or slow search speed in later stages when dealing with complex functions.

To address these issues, a multi-strategy improved PKO algorithm (MSIPKO) is proposed in this study. The core idea of MSIPKO is to enhance the balance between global exploration and local exploitation through the integration of multiple complementary strategies.

Specifically, three improvement strategies are introduced. First, a reverse differential crossover mechanism is incorporated to compensate for the lack of mutation in the original PKO and enhance population diversity. Second, during the symbiotic stage, a detour-foraging mechanism inspired by zebrafish behavior is embedded into the second branch of Formula (14) to improve the search capability during the commensalism phase. Finally, a diving-foraging module inspired by pied kingfisher behavior is introduced to strengthen the search efficiency and maintain convergence performance in later iterations.

Through the integration of these strategies, MSIPKO is able to achieve a better balance between exploration and exploitation, thereby improving convergence stability and optimization accuracy. The detailed formulations of the proposed strategies are presented in the following subsections.

### 3.2. Improvement Strategies

#### 3.2.1. Reverse Differential Crossover Mechanism

In this operator, first, reverse learning is used to simulate the process of reverse searching for food in pied kingfisher, and the calculation formula is shown in Formula (16).

(16) $$X_{i}^{op}\left(t\right)=LB+UB-X_{i}\left(t\right)$$

where $X_{i}^{op}\left(t\right)$ represents the inverse solution after the *t*-th iteration.

Subsequently, after merging the original population and the reverse population, differential mutation operations are performed on the resulting population $X^{new}\left(t\right)$. The specific calculation process is shown in Formulas (17) and (18). This step simulates the process in which pied kingfishers expand their search range and interact with each other within the group to exchange information.

(17) $$X_{i}^{v}\left(t\right)=X_{i}^{new}\left(t\right)+rand\left(1,\dim\right)\cdot \left[X_{i}^{new}\left(t\right)-X_{k}^{new}\left(t\right)\right]$$

(18) $$\begin{array}{l} X_{i,j}\left(t+1\right)=\left\{\begin{array}{l} X_{i,j}^{new}\left(t\right),rand\leq 0.5\\ X_{i,j}^{v}\left(t\right),rand>0.5 \end{array}\right.\\ i=1,2,\ldots ,N\;\;\;\;\;\;\;\;\;\;\;\;\;j=1,2,\ldots ,\dim \end{array}$$

where $X_{i}^{new}\left(t\right)$ and $X_{k}^{new}\left(t\right)$ are the *i*-th and *k*-th individuals after population merging, respectively. $rand\left(1,\dim\right)$ means a random vector with the range of [0, 1] of length dimensions.

#### 3.2.2. Enhanced Diving-Fishing Operator

An enhanced operator, as defined in Formulas (19)–(21), is proposed to improve the search efficiency of the pied kingfisher’s diving behavior during the prey-capturing process in the PKO algorithm.

(19) $$X_{i}\left(t+1\right)=\lambda_{1}\cdot X_{i}\left(t\right)-\lambda_{2}\cdot X_{rand\text{\_}ind}\left(t\right)+\lambda_{3}\cdot X_{step}\left(t\right)$$

(20) $$X_{step}\left(t\right)=\left(UB-LB\right)\cdot \lambda_{4}$$

(21) $$\lambda_{4}=\;\;\exp\left[0.1\cdot \dim\cdot {\left(\frac{t}{Max\text{\_}Iter}\right)}^{2}-\frac{0.2\cdot \dim\cdot t}{Max\text{\_}Iter}\right]$$

where $\lambda_{1},\lambda_{2},\lambda_{3}$ are three random numbers within the interval [0, 1]. $X_{rand\text{\_}ind}\left(t\right)$ represents any individual in the population other than $X_{i}$ itself in *t*-th iteration. $X_{step}$ is a disturbance variable.

#### 3.2.3. Improvement of the Commensalism Phase

To enhance the search performance of the PKO algorithm, the second branch of Formula (14) was improved to address the issue where individuals might not search toward a better direction. The modified formula is presented as Formulas (22)–(25).

(22) $$X_{i}\left(t+1\right)=\left\{\begin{array}{l} X_{m}\left(t\right)+ο\cdot \alpha \cdot \left|X_{i}\left(t\right)-X_{n}\left(t\right)\right|,\;\;rand>1-PE\\ X_{rand\text{\_}ind}\left(t\right)+\lambda_{5},\;\;\;\;\;\;\;\;\;\;\;\;\;\;\;\;\;\;\;\;\;\;\;\;rand\leq 1-PE \end{array}\right.$$

(23) $$\lambda_{5}=R\cdot \left[X_{i}\left(t\right)-X_{rand\text{\_}ind}\left(t\right)\right]+\delta$$

(24) $$R=\left[\exp\left(1\right)-\exp{\left(\frac{t-1}{Max\text{\_}Iter}\right)}^{2}\right]\cdot \sin\left(2\pi \cdot rand\right)$$

(25) $$\delta =round\left[0.5\cdot \left(0.05+rand\right)\right]\cdot randn\left(1,\dim\right)$$

where *R* is a zoom factor. δ is a disturbance variable. *round* means round to the nearest integer.

### 3.3. The Flow of MSIPKO

MSIPKO is designed as a unified hybrid optimization framework, as shown in Algorithm 2, in which three carefully selected strategies function in a complementary and coordinated manner. This integration is not a simple stacking of heuristics, but a structured synergy aimed at addressing key limitations in the original PKO algorithm. Specifically, the reverse differential crossover enhances global exploration in early stages, the enhanced diving-fishing operator improves local exploitation during convergence, and the improved commensalism phase ensures guided adaptation in later iterations. These strategies work together to improve search diversity, convergence speed, and solution accuracy under complex constraints. Based on this integrated framework, the complete pseudocode of MSIPKO is presented below to illustrate its iterative process.
**Algorithm 2: Pseudocode of MSIPKO****Input:** Population total number *N*; the number of optimization iterations *MaxIt*. **Output:** The optimal solution of PKO (Best_fitness), optimal solution vector *X*_best_.**1****Initialize** the PKO population positions according to Section 2.1.**2**Calculate the pied kingfisher fitness values**3****while** (*t* < *MaxIt* + 1) **do****4**  **for** i = 1:*N* **do****5**     Implement reverse differential crossover mechanism according to Formulas (16)–(18).**6**     If the newly generated solutions are superior to the previous ones, then replace them. Set best position as the location of best fitness.**7**         **if** (rand < 0.8) **then****8**      **% Exploration phase****9**      **if** (rand > 0.5) **then****10**        Compute *T*_1_ according to Formulas (6) and (7)**11**        Update the position of pied kingfisher using Formula (4)**12**      **else****13**        Compute *T*_2_ according to Formulas (8) and (9)**14**        Update the position of pied kingfisher using Formula (4)**15**     **end****16**   **else****17**    **% Exploitation phase****18**      Update the position of pied kingfisher using Formula (10)**19**    **end****20**    If the newly generated solutions are superior to the previous ones, then replace them. Set best position as the location of best fitness.**21**     **if** (rand > (1 − *PE*)) **then****22**      Update the position of pied kingfisher using the first branch of Formulas (14) and (15)**23**    **else****24**      Update the position of pied kingfisher using Formulas (19)–(21)**25**   **end****26**   Calculate the fitness values of pied kingfisher**27**    If the newly generated solutions are superior to the previous ones, then replace them. Set best position as the location of best fitness.**28**   **% Local escape phase****29**   Implement commensalism phase according to Formulas (22)–(25) and (15).**30**   If the newly generated solutions are superior to the previous ones, then replace them. Set best position as the location of best fitness.**31**   *t* = *t* + 1**32**  **end for****33****end while****34**Return Best_fitness and *X*_best_.

### 3.4. Computational Complexity of MSIPKO Analysis

To assess the computational efficiency of the proposed MSIPKO algorithm, this section compares its theoretical time complexity with the original PKO algorithm. Both algorithms operate under the same population size *N*, problem dimension *D*, and maximum number of iterations *T*.

Table 2 summarizes the time complexity of key modules in both algorithms. Structurally, PKO performs three stages per generation—hovering/perching, diving, and commensalism—each involving $O\left(N\cdot D\right)$ scale updates and evaluations. Therefore, the complexity for all iterations is $O\left(3\cdot T\cdot N\cdot D\right)$. Considering the initialization, the total complexity is $O\left[\left(3\cdot T+1\right)\cdot N\cdot D\right]$.

On top of retaining these three stages, MSIPKO introduces one additional module: the reverse differential crossover mechanism, which involves generating reverse individuals and applying differential mutation, contributing an extra $O\left(N\cdot D\right)$ computational load per generation. It is important to emphasize that the enhanced diving-fishing operator and the improved commensalism phase in MSIPKO are refined versions of the original PKO operators. These enhancements only adjust local update rules without introducing new loops or global operations, thus do not increase the asymptotic complexity. Consequently, MSIPKO’s per-generation complexity is $O\left(4\cdot T\cdot N\cdot D\right)$, and total complexity is $O\left[\left(4\cdot T+1\right)\cdot N\cdot D\right]$. While the constant factor increases slightly, the overall asymptotic order remains unchanged. It should be noted that the above analysis focuses on asymptotic time complexity, whereas the additional module mainly affects the constant computational cost per iteration rather than the overall complexity order.

In addition to the asymptotic time complexity analysis, the computational burden of MSIPKO is further evaluated from a practical perspective. Although the additional module increases the constant computational cost per iteration, the experimental results indicate that MSIPKO generally requires fewer function evaluations to achieve comparable or better solution quality than most competing algorithms. This suggests that the im proved search efficiency effectively compensates for the additional computational overhead.

Therefore, MSIPKO maintains a reasonable computational burden while achieving improved optimization performance, demonstrating a favorable trade-off between computational cost and solution quality. Table 2 presents a module-level comparison of computational cost, reflecting the relative overhead of each component rather than the asymptotic complexity order.

### 3.5. Experimental Settings and Constraint-Handling Strategy

To ensure a fair and reliable comparison, all experiments are conducted under a unified experimental setting.

To ensure a fair and reliable comparison, all algorithms are implemented in MATLAB R2024a and executed on a macOS-based system equipped with an Apple M3 Pro processor and 18 GB RAM. For each test problem, all algorithms are independently run 30 times to reduce the influence of randomness.

For constrained optimization problems, a static penalty function is adopted. The penalized objective function is defined as Equation (26).

(26) F(x)=f(x)+ω⋅G(x)

where F(x) is the fitness function, $f\left(x\right)$ is the original objective function, and G(x) is called the violation function, whose calculation process is shown in Equation (2). ω is the penalty coefficient. In this study, ω is set to 10^6^ for all constrained problems. This formulation ensures that infeasible solutions are strongly penalized and guides the population toward the feasible region during the optimization process.

All compared algorithms use the parameter settings recommended in their original studies to ensure fairness of comparison.

## 4. Numerical Experiment

This section presents a comprehensive set of experiments to evaluate the performance of the proposed MSIPKO algorithm on constrained optimization problems (COPs). The evaluation is divided into two parts: benchmark problems from the CEC 2006 suite (G01–G12) and six classical engineering design problems.

For the benchmark tests, MSIPKO is compared against nine representative algorithms, including recent methods such as the enzyme action optimizer (EAO) and the starfish optimization algorithm (SFOA), forming a set of ten algorithms in total. All algorithms are tested under unified simulation conditions and use a static penalty function as the default constraint-handling mechanism. Performance is assessed through metrics such as average fitness, best solution, worst solution, and standard deviation. Statistical significance is verified using Wilcoxon signed-rank tests with Bonferroni correction.

Next, a component-wise ablation study is conducted to assess the contribution of each improvement in MSIPKO, by sequentially integrating the proposed strategies into the baseline PKO. Following that, a sensitivity analysis under varying evaluation budgets is performed, examining the algorithm’s ability to converge under six different function evaluations (FEs) limits, ranging from 1000 to 200,000.

Furthermore, a detailed comparison of different constraint-handling strategies (CHMs) is presented. MSIPKO is embedded within multiple CHMs, including static penalty, dynamic penalty, feasibility rules, ε-constrained method, and equality relaxation. This comparison highlights the algorithm’s robustness across diverse constraint environments.

In the second part, six widely studied engineering problems are used to further validate the real-world applicability of MSIPKO. The results confirm its strong and stable performance under practical design constraints.

### 4.1. Benchmark Functions

In this section, the performance of the proposed MSIPKO algorithm is thoroughly examined. Twelve benchmark functions are used to evaluate its optimization ability, and ten state-of-the-art algorithms are employed for comparison. This section includes five parts: (1) introduction of benchmark functions; (2) simulation setup and statistical analysis; (3) component-wise ablation study of MSIPKO; (4) sensitivity to evaluation budget; and (5) comparison of constraint-handling strategies.

#### 4.1.1. Introduction of Benchmark Functions

The main characteristics of the 12 functions are shown in Table 3. Among them, **n** is the number of decision variables. **ρ** is the estimated ratio between the feasible region and the search space. LI is the number of linear inequality constraints. NI is the number of nonlinear inequality constraints. LE is the number of linear equality constraints, and NE is the number of nonlinear equality constraints. a is the number of active constraints.

#### 4.1.2. Comparative Evaluation of 10 Algorithms: Simulation Setup and Statistical Analysis

(1) Simulation settings

In order to verify MSIPKO’s performance, eight algorithms that have shown high quality in the last two years are compared, including PKO, flood algorithm (FLA) [32], black-winged kite algorithm (BKA) [33], triangulation topology aggregation optimizer (TTAO) [34], FOX-inspired optimization algorithm (FOX) [35], Crayfish optimization algorithm (COA) [36], Newton–Raphson-based optimizer (NRBO) [37], enzyme action optimizer (EAO) [38] and starfish optimization algorithm [7].

In order to ensure the fairness of numerical simulation, these 10 algorithms have consistent simulation conditions when calculating 12 benchmark functions by executing 30 times independently. The specific parameters and constraint processing strategies for numerical simulation are shown in Table 4, FEs represent function evaluations, which is population size multiplied by iterations. The relevant parameters of each algorithm are consistent with their proposed literature.

(2) Experimental results

**Figure 1:**
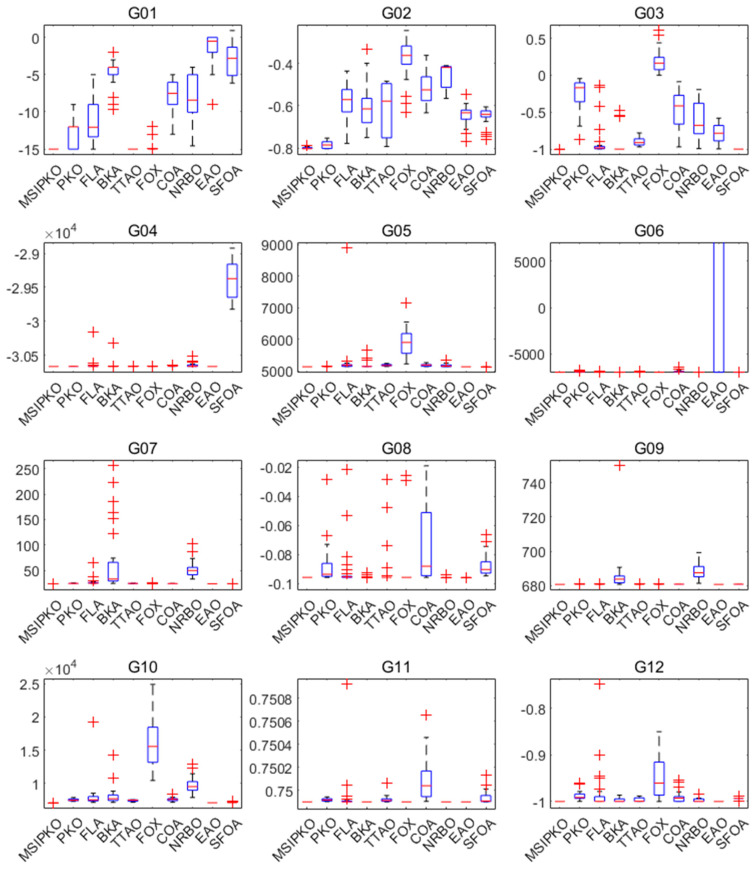
Boxplot of MSIPKO and 9 competitor algorithms for 12 benchmark functions of CEC 2006.

According to the simulating condition in Table 4, the performance of G01–G12, including the mean value, standard deviation, and the best and worst value, is summarized in Table 5 and Table 6 and visualized through the box plots in Figure 1. Specifically, Table 4 presents the results of the first five algorithms, while Table 5 shows those of the remaining five algorithms.

From the numerical results, MSIPKO demonstrates a strong ability to consistently reach near-optimal or best-known solutions in most test cases. Specifically, on G01, G02, G06, and G08, the algorithm shows both low standard deviation and excellent best-case performance, indicating reliable convergence behavior. In constrained equality-dominant problems such as G03 and G07, MSIPKO still maintains competitive quality with relatively tight result distributions.

Figure 1 complements this analysis by highlighting the solution distributions of all algorithms. MSIPKO exhibits compact box ranges on most problems, reflecting high stability. For functions like G04 and G09, although multiple algorithms show feasible outputs, the interquartile range of MSIPKO is narrower, with fewer outliers. This suggests the multi-strategy enhancement successfully mitigates population stagnation and maintains solution diversity in high-constraint scenarios.

Overall, the simulation results validate the effectiveness of MSIPKO across different categories of constrained optimization problems.

To further validate the statistical significance of the non-parametric statistical tests was conducted. The next subsection presents the ranking results, Wilcoxon signed-rank test outcomes, and Bonferroni-corrected significance analysis to confirm the robustness and reliability of MSIPKO’s superiority.

In the present study, a static penalty function with a penalty factor of 10^6^ is adopted for both the CEC 2006 benchmark problems and the engineering design problems. Under this setting, infeasible solutions would result in substantially inflated objective values. From the reported results, most algorithms obtain objective values within the expected feasible range on the majority of problems, indicating that they generally reach the feasible region or remain very close to it. A notable exception is EAO on G06, where the unusually large objective values suggest possible difficulty in reaching feasible solutions in some runs.

(3) Statistical analysis

To further validate the algorithm’s superiority and robustness, statistical analyses including rank-based comparison and non-parametric Wilcoxon signed-rank test with Bonferroni correction were conducted on the results across all benchmark functions.

As shown in Table 7, the proposed MSIPKO algorithm achieves rank 1 on most benchmark functions, and secures rank 2 on a few functions. While the EAO ranks first on some functions including G05, G07, G09, etc., its performance significantly deteriorates on others (e.g., G01–G03, G06), resulting in an overall mean rank of 3.33, which is inferior to MSIPKO (mean rank = 1.42). This indicates that EAO may exhibit strength in specific local patterns but lacks general applicability. In contrast, MSIPKO demonstrates stable and universal performance, suggesting its broader suitability for solving diverse constrained optimization problems.

Furthermore, other algorithms such as PKO, BKA, TTAO, and FOX present relatively fluctuating ranks, showing inconsistent effectiveness depending on the problem structure. Notably, the original PKO ranks much lower (mean rank = 5.92), highlighting the significant improvements gained through the enhancements introduced in MSIPKO.

To further validate the statistical significance of the observed performance advantages, a Wilcoxon signed-rank test is performed between MSIPKO and each of the other nine algorithms. Additionally, the Bonferroni correction was applied to adjust the significance levels in the context of multiple comparisons. The detailed results of these non-parametric statistical tests are presented in Table 7.

As shown in Table 8, the Wilcoxon signed-rank test is employed to assess the statistical significance of performance differences between MSIPKO and the comparison algorithms. For each benchmark function, all algorithms are independently executed 30 times, and the mean objective value is used as the representative performance metric.

For each pair of algorithms, a paired sample is constructed by comparing their mean objective values on each benchmark function. That is, each function provides one paired data point, and the Wilcoxon signed-rank test is conducted across all benchmark functions. This pairing scheme enables a fair comparison by evaluating algorithm performance consistently on the same set of problem instances.

The significance level is set to α = 0.05, and the Bonferroni correction is applied with m = 9, representing the total number of pairwise comparisons between MSIPKO and each of the nine competing algorithms, to control the family-wise error rate.

The results indicate that MSIPKO generally achieves better performance compared to most of the considered algorithms. For PKO, FLA, BKA, TTAO, FOX, COA, NRBO, and SFOA, the obtained *p*-values (e.g., 0.0005) indicate statistically significant differences at the 0.05 significance level before correction, and these differences remain significant after applying the Bonferroni correction.

For EAO, the *p*-value is 0.0312, indicating relatively weaker statistical evidence. After Bonferroni correction, the adjusted *p*-value (0.2808) does not meet the significance thresholds. However, the rank-sum results (R+ > R−) still suggest that MSIPKO tends to outperform EAO in most cases.

Overall, these results suggest that MSIPKO demonstrates competitive and generally superior performance compared with the majority of the considered algorithms, while maintaining statistical robustness under multiple comparison conditions.

#### 4.1.3. Component-Wise Ablation Study of MSIPKO

**Figure 2:**
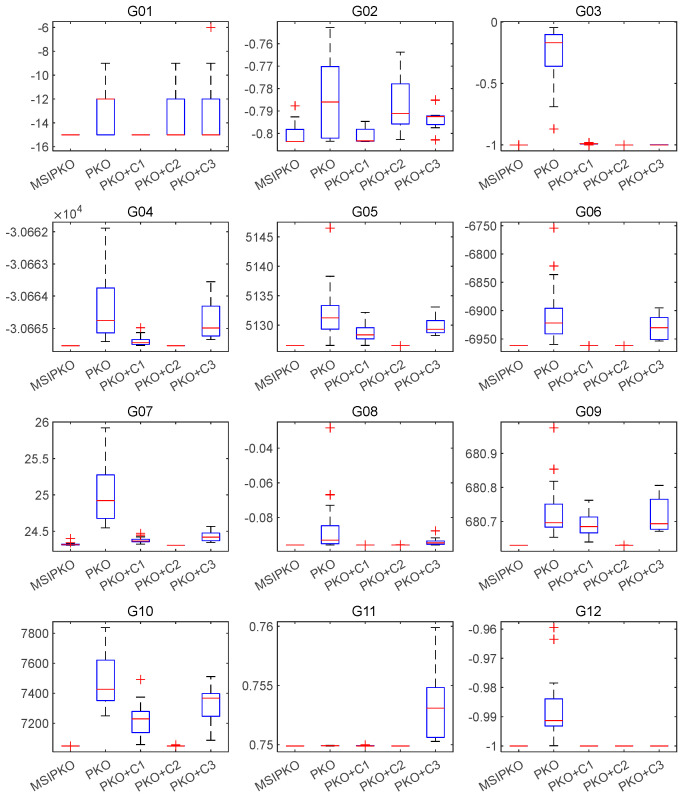
Boxplot of MSIPKO’s component-wise ablation study for 12 benchmark functions of CEC 2006.

To further validate the effectiveness of each improvement component in the MSIPKO algorithm, this section conducts an ablation study involving five variants: the original PKO, PKO enhanced with Component 1 (PKO + C1), Component 2 (PKO + C2), Component 3 (PKO + C3), and the full version MSIPKO, where C1 denotes the reverse differential crossover mechanism, C2 represents the enhanced diving-fishing operator, and C3 introduces the improvement of the commensalism phase. The comparative results across 12 benchmark functions are summarized in Table 9 and visualized in Figure 2 using boxplots. Additionally, a ranking-based evaluation is conducted in Table 10 to assess the effectiveness of each component and their synergy.

From the data in Table 10, MSIPKO achieves the best performance on 10 out of the 12 benchmark functions, and ranks second on the remaining two (G02 and G07), resulting in the lowest total ranking score with the number 14 and an average rank of 1.17. This indicates that the integration of all three components leads to consistently superior performance and robustness. By contrast, the original PKO ranks worst overall (average rank = 4.83), confirming the necessity of component enhancements.

Detailed analysis reveals the individual and synergistic contributions of each component. Component C1 plays a decisive role in improving the optimization accuracy for functions G01 and G02. Component C2 dominates the improvements in G04, G05, G07, G09, and G10, showcasing its ability to enhance convergence and robustness in complex constrained scenarios. Component C3, though not solely responsible for the best results on any single function, contributes a stabilizing effect, particularly in the combination setting.

Moreover, some benchmark functions benefit from the synergy of multiple components. Specifically, G03 and G12 are significantly improved only when all three components are integrated, with the most notable contribution in G03 coming from C3. Functions G06, G08, and G11 are enhanced primarily by the combined effect of C1 and C2, indicating their complementary strengths.

It is worth noting that in the cases of G02 and G07, MSIPKO performs slightly worse than the single-module-enhanced versions (PKO + C1 and PKO + C2, respectively). This is likely due to the slight trade-off effect introduced by full-component integration, where the balancing mechanism of C3 may suppress the localized strength of individual modules in specific scenarios.

Overall, the results from Table 9 and Table 10 and Figure 2 demonstrate that each component contributes uniquely to the algorithm. The combined use of C1, C2, and C3 in MSIPKO ensures both high accuracy and strong stability, making it a robust and well-rounded approach for constrained optimization problems.

#### 4.1.4. Sensitivity to Evaluation Budget

**Figure 3:**
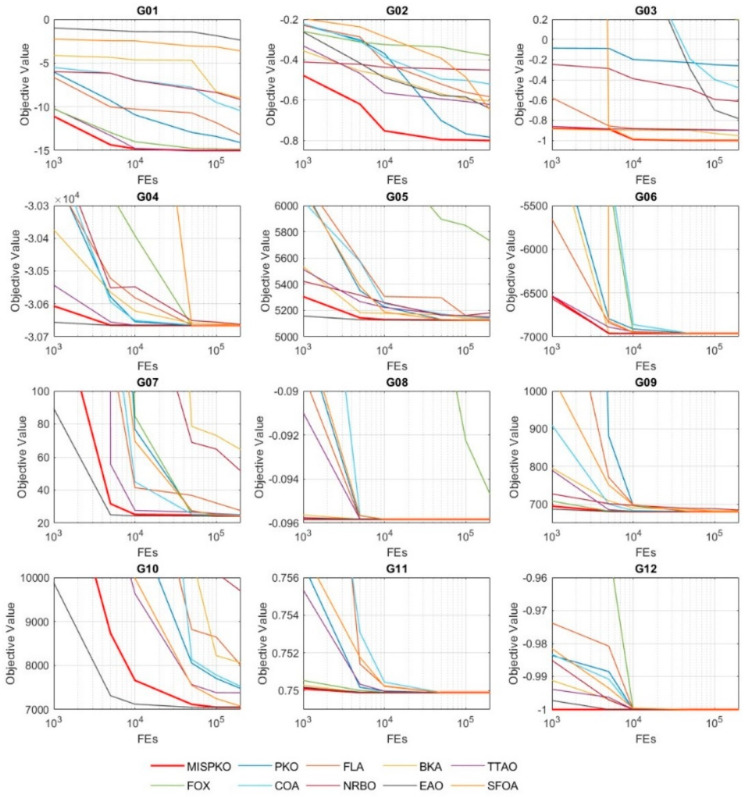
Sensitivity analysis of 10 algorithms under varying FEs.

To further evaluate the response sensitivity and efficiency of the 10 algorithms under varying computational budgets, this section analyzes the average objective values across six levels of function evaluations, including 1000; 5000; 10,000; 50,000; 100,000; 200,000, as visualized in Figure 3.

When the number of function evaluations (FEs) is less than 10,000, most algorithms—such as PKO, FLA, and FOX—exhibit relatively slow convergence, with noticeable gaps between initial and near-optimal values. In contrast, some algorithms, including EAO, BKA, and FLA, show relatively faster descent on certain functions (e.g., G02, G07, and G09). Among them, EAO often demonstrates rapid improvement at the early stage for several functions, although its subsequent progress becomes limited in some cases (e.g., G02 and G03). Similarly, BKA shows good initial performance but tends to plateau relatively early.

In comparison, MSIPKO exhibits efficient early-stage convergence behavior, achieving objective values close to the optimum on several functions (e.g., G01, G02, G06, and G12) within FEs = 10,000.

As the evaluation budget increases to FEs = 50,000 and beyond, the performance differences become more apparent. MSIPKO continues to improve its solutions and generally maintains lower objective values on most functions. For example, on G02, MSIPKO gradually surpasses other algorithms. In the later stages, FOX and COA demonstrate relatively better fine-tuning ability on some functions (e.g., G03, G05, and G10), suggesting stronger local exploitation capability, although their overall performance remains inferior to MSIPKO.

Overall, MSIPKO demonstrates competitive and generally superior convergence behavior across most test functions. The convergence curves exhibit relatively smooth trends in many cases, although some fluctuations may still be observed. No obvious premature convergence is observed in most functions. These results suggest that MSIPKO achieves a reasonable balance between global exploration and local exploitation under different computational budgets.

#### 4.1.5. Comparison of Constraint-Handling Strategies

To evaluate the performance of MSIPKO in comparison with algorithms utilizing different constraint-handling strategies, this study conducts a comparative analysis against five representative algorithms. These algorithms adopt various constraint-handling approaches, including the HMICA [1] based on Deb’s criterion, the BSA-SAε algorithm [39] employing the ε-constrained method, and three algorithms—SMA-GM [40], AGWO [41], and IChoA [42]—using the dynamic penalty approach.

The simulation conditions and constraint-handling strategies for these algorithms are summarized in Table 11, and the corresponding results are presented in Table 12. It should be emphasized that the results in Table 12 are partially collected from the literature, where different algorithms were evaluated under different function evaluation (FE) budgets. Therefore, these results should be interpreted as indicative comparisons rather than strictly controlled evaluations.

As shown in Table 12, MSIPKO demonstrates competitive performance compared with the other algorithms. However, some algorithms exhibit slightly better reported average or optimal values for certain benchmark functions, such as G04, G05, G06, G08, and G11. These discrepancies are mainly attributed to differences in numerical precision and rounding conventions across studies rather than intrinsic performance differences.

For example, for the G04 benchmark function, the theoretical optimum is reported as $f\left(x^{*}\right)=\text{−}30,665.53867178332$ in the CEC 2006 benchmark definition, while some studies present rounded values such as −30,665.539 or even −30,666.

According to the official CEC 2006 evaluation criteria, solution quality is assessed based on function error rather than decimal precision. Specifically, a solution is considered successful if $f\left(x\right)-f\left(x^{*}\right)\leq 10^{-4}$ while satisfying feasibility conditions.

Therefore, in this study, solutions that satisfy this error threshold are regarded as equivalent to the theoretical optimum. This criterion ensures consistency with the benchmark evaluation protocol and avoids misleading comparisons caused by differences in reported decimal precision.

Another important issue is the inconsistency in FE budgets. Specifically, the three algorithms employing dynamic penalty strategies were evaluated under a fixed FE budget of 30,000 in their original studies, which is lower than that used by MSIPKO for several benchmark functions (G01, G02, G03, G05, G07, G09, and G10). To ensure a fairer comparison, MSIPKO was additionally evaluated under the same FE budget (30,000) for these functions, and the results are presented in Table 13.

Compared with Table 12, Table 13 provides a more reliable and directly comparable assessment under unified computational conditions. Based on these results, MSIPKO maintains competitive performance under equal evaluation budgets.

Given the aforementioned scenarios, a simple comparison using average rankings or the Wilcoxon test is not suitable when evaluating against studies that adopt different constraint-handling strategies. Instead, a detailed comparison of the results obtained by MSIPKO and other algorithms on each benchmark function is necessary. Additionally, when MSIPKO and another algorithm reach the theoretical optimal value for a benchmark function, the precision retained in the literature is disregarded. In this case, the results of the two algorithms are considered equivalent.

The comparison rules are as follows:

(1) If the results of both algorithms are identical, their performance is further compared based on the number of function evaluations, with the algorithms requiring fewer evaluations being deemed superior.
(2) If one algorithm achieves better results while requiring no more function evaluations than the other, it is considered to have superior performance. In this study, MSIPKO demonstrates that its number of function evaluations for all benchmark functions (G01–G12) is consistently less than or equal to that of the comparison algorithms.
(3) If one algorithm outperforms the other in the comparison, it is marked with a “+”; if the other method is superior, it is marked with a “−”; and if both methods yield equivalent results, it is marked with an “=”.

Table 14 presents the comparison results of MSIPKO and five other algorithms across 12 benchmark functions. It should be noted that the results presented in Table 14 are derived based on a combination of the results from Table 12 and Table 13. For benchmark functions with consistent function evaluation settings, results from Table 13 are used, while results from Table 12 are adopted for the remaining functions. Combined with the data and analysis rules discussed earlier, MSIPKO shows competitive performance with consistent advantages across most benchmark functions.

First, MSIPKO achieved 53 positive outcomes (“+” marks), 3 ties (“=” marks), and 4 negative outcomes (“−” marks) in the comparisons. Its overall performance across the 12 benchmark functions outperformed other algorithms, particularly in nonlinear complex constrained problems where it showcased exceptional optimization capabilities. For instance, in complex functions such as G02 and G10, MSIPKO not only achieved the theoretical optimal values but also significantly reduced the number of function evaluations (up to 200,000, compared to 350,000 for BSA-SAε), achieving an excellent balance between efficiency and accuracy.

In the comparison with HMICA, MSIPKO performed better on most benchmark functions, with tied results on G04, G08, and G12 (“=” marks), highlighting its superior efficiency and robustness. Against BSA-SAε, MSIPKO outperformed in all benchmark functions, demonstrating its strong adaptability to different types of constraints. While SMA-GM showed slightly better performance on G01 and G03, MSIPKO surpassed it on all other benchmark functions. Although MSIPKO showed a slight disadvantage in G03 when fewer function evaluations were used compared to algorithms employing dynamic penalty functions, results with 200,000 function evaluations showed that MSIPKO still achieved the theoretical optimal value, indicating its competitive search precision. Particularly in complex constrained problems such as G05, G07, and G09, MSIPKO obtained superior solutions with fewer function evaluations. Additionally, MSIPKO significantly outperformed AGWO and IChoA, especially in nonlinear problems, where its mean and optimal values were markedly better, further emphasizing its precision advantages.

In summary, MSIPKO demonstrated computational efficiency, robustness, and optimization capabilities in the benchmark tests, validating its exceptional performance as an effective constrained optimization algorithm.

### 4.2. Engineering Problems

The method proposed in this paper addresses six common engineering optimization problems, including the I-beam vertical deflection problem, the speed reducer design problem, the three-bar truss design problem, the welded beam design problem, the tension/compression spring design problem, and the pressure vessel design optimization problem. The performance of MSIPKO is validated by comparing its results with those of nine other algorithms across these problems.

This section is divided into two parts. The first part provides a brief introduction to the six engineering problems, while the second part presents the computational results of various algorithms and offers an analysis and discussion of their performance.

#### 4.2.1. Introduction of Six Engineering Problems

(1) I-beam vertical deflection problem

The I-beam vertical deflection problem is a classic engineering optimization problem. The objective is to minimize the vertical deflection of the I-beam by optimizing geometric parameters such as length ($x_{1}$), height ($x_{2}$), web thickness ($x_{3}$), and flange thickness ($x_{4}$), while satisfying constraints on cross-sectional area and material stress. This problem is characterized by its constrained and nonlinear nature, requiring a balance between structural strength, stiffness, and material efficiency, which adds to its computational complexity. The design variables, including length, height, web thickness, and flange thickness, each have a significant impact on the structural performance.

I-beams are widely used in construction, mechanical engineering, and transportation, such as in bridges, floors, equipment frames, and railway tracks. Optimizing the design of I-beams not only reduces material consumption and costs but also enhances load-bearing capacity and structural safety. However, the problem involves complex constraints that require comprehensive consideration of strength, stiffness, and stability. Additionally, the optimization process often exhibits nonlinear and multimodal characteristics, increasing the difficulty of finding solutions.

The mathematical formulation of this problem is given in Formulas (27)–(29).

(27) $$\min\;\;f\left(x\right)=\frac{5000}{\frac{x_{3}\cdot \left(x_{2}-2\cdot x_{4}\right)}{12}+\frac{x_{1}\cdot x_{4}^{3}}{6}+x_{1}\cdot x_{4}\cdot \frac{{\left(x_{2}-x_{4}\right)}^{2}}{2}}$$

*s.t.*

(28) $$g_{1}\left(x\right)=2\cdot x_{1}\cdot x_{4}+x_{3}\cdot \left(x_{2}-2\cdot x_{4}\right)-300\leq 0$$

(29) $$g_{2}\left(x\right)=\frac{18\times 10^{4}\cdot x_{2}}{x_{3}\cdot y^{3}+2\cdot x_{1}\cdot x_{3}\cdot \left(4\cdot x_{4}^{2}+3\cdot x_{2}\cdot y\right)}\;\;+\frac{15\times 10^{3}\cdot x_{1}}{x_{3}^{3}\cdot y+2\cdot x_{1}^{3}\cdot x_{3}}-56\leq 0$$

where $y=x_{2}-2\cdot x_{4}$, $10\leq x_{1}\leq 50$, $10\leq x_{2}\leq 80$, $0\leq x_{3},x_{4}\leq 9.5$.

(2) Speed reducer design problem

The speed reducer is a critical component in mechanical transmission systems and is widely used in industrial machinery, automotive power systems, and energy equipment such as wind turbines. It serves to regulate speed and transmit torque. Optimizing the design of speed reducers not only helps reduce their manufacturing cost and weight but also significantly enhances their efficiency and reliability.

This problem represents a classic constrained mixed-integer optimization challenge, where the primary difficulty lies in achieving the optimal balance between performance and weight while addressing the computational complexities introduced by nonlinear constraints and mixed variables. The objective is to minimize the weight of the speed reducer while satisfying mechanical performance constraints. These constraints primarily involve gear bending stress, surface contact stress, lateral deflection of the shaft, and internal stress within the shaft. The problem involves seven design variables: gear width ($x_{1}$), module ($x_{2}$), number of pinion teeth ($x_{3}$), length of the first shaft ($x_{4}$), length of the second shaft ($x_{5}$), diameter of the first shaft ($x_{6}$), and diameter of the second shaft ($x_{7}$). These variables include both continuous variables (e.g., shaft diameters) and discrete variables (e.g., number of teeth), further increasing the complexity of the problem. For the speed reducer design problem, the variable $x_{3}$, representing the number of pinion teeth, is treated as an integer variable. In this study, an integer constraint is imposed on $x_{3}$ during function evaluation and result reporting to ensure consistency with the original problem formulation.

The mathematical formulation of this problem is given in Formulas (30)–(41).

(30) $$\begin{array}{l} \min\;\;f\left(x\right)=0.7854\cdot x_{1}\cdot x_{2}^{2}\cdot \left(3.3333\cdot x_{3}^{2}+14.9334\cdot x_{3}\right.\;\left.-43.0934\right)-1.508\cdot x_{1}\cdot \left(x_{6}^{2}+x_{7}^{2}\right)\;+7.4777\cdot \\ \;\;\;\;\;\;\;\;\;\;\;\;\;\;\;\;\;\;\;\;\;\;\;\;\;\;\;\;\;\left(x_{6}^{3}+x_{7}^{3}\right)+0.7854\cdot \left(x_{4}\cdot x_{6}^{2}+x_{5}\cdot x_{7}^{2}\right) \end{array}$$

*s.t.*

(31) $$g_{1}\left(x\right)=\frac{27}{x_{1}\cdot x_{2}^{2}\cdot x_{3}}-1\leq 0\;\;$$

(32) $$g_{2}\left(x\right)=\frac{397.5}{x_{1}\cdot x_{2}^{2}\cdot x_{3}^{2}}-1\leq 0$$

(33) $$g_{3}\left(x\right)=\frac{1.93\cdot x_{4}^{3}}{x_{2}\cdot x_{3}\cdot x_{6}^{4}}-1\leq 0$$

(34) $$\;g_{4}\left(x\right)=\frac{1.93\cdot x_{5}^{3}}{x_{2}\cdot x_{3}\cdot x_{7}^{4}}-1\leq 0$$

(35) $$g_{5}\left(x\right)=\frac{1}{110\cdot x_{6}^{3}}\cdot \sqrt{{\left(\frac{745\cdot x_{4}}{x_{2}\cdot x_{3}}\right)}^{2}+16.9\times 10^{6}}-1\leq 0$$

(36) $$g_{6}\left(x\right)=\frac{1}{85\cdot x_{7}^{3}}\cdot \sqrt{{\left(\frac{745\cdot x_{5}}{x_{2}\cdot x_{3}}\right)}^{2}+157.5\times 10^{6}}-1\leq 0$$

(37) $$g_{7}\left(x\right)=\frac{x_{2}\cdot x_{3}}{40}-1\leq 0$$

(38) $$g_{8}\left(x\right)=\frac{5\cdot x_{2}}{x_{1}}-1\leq 0$$

(39) $$g_{9}\left(x\right)=\frac{x_{1}}{12\cdot x_{2}}-1\leq 0\;$$

(40) $$g_{10}\left(x\right)=\frac{1.5\cdot x_{6}+1.9}{x_{4}}-1\leq 0$$

(41) $$g_{11}\left(x\right)=\frac{1.1\cdot x_{7}+1.9}{x_{5}}-1\leq 0\;\;\;\;\;\;$$

where $2.6\leq x_{1}\leq 3.6$, $0.7\leq x_{2}\leq 0.8$, $17\leq x_{3}\leq 28$, $7.3\leq x_{4},x_{5}\leq 8.3$, $2.9\leq x_{6}\leq 3.9$, $5\leq x_{7}\leq 5.5$.

(3) Three-bar truss design problem

The three-bar truss design problem is a structural optimization problem with wide-ranging applications. In construction engineering, three-bar trusses are commonly used in bridges, roofs, and high-rise buildings, where optimized designs improve material utilization and reduce costs. In mechanical manufacturing, lightweight truss structures are applied to machine supports and frames, reducing weight while maintaining strength. In aerospace engineering, optimizing truss designs is crucial for reducing the structural weight of aircraft and spacecraft while enhancing their performance.

The three-bar truss design problem is a classic structural optimization problem. The objective is to minimize the total volume of the three-bar truss by optimizing its cross-sectional areas while satisfying the bearing capacity constraints of each truss member. In this problem, the objective function represents the total material volume of the truss, calculated as the sum of the products of the cross-sectional areas and the lengths of the truss members. The constraints ensure that the truss satisfies the required bearing capacity and structural stability while achieving volume minimization.

The main challenge of the three-bar truss design problem lies in handling the complex nonlinear constraints and interdependencies among variables, while achieving an optimal balance between strength, stability, and volume minimization. Its mathematical formulation is provided in Formulas (42)–(45).

(42) $$\min\;\;f(x)=\left(2\sqrt{2}\cdot x_{1}+x_{2}\right)\times l$$

*s.t.*

(43) $$g_{1}(x)=\frac{\sqrt{2}\cdot x_{1}+x_{2}}{\sqrt{2}\cdot x_{1}^{2}+x_{1}\cdot x_{2}}\cdot P-\sigma \leq 0$$

(44) $$g_{2}(x)=\frac{x_{2}}{\sqrt{2}\cdot x_{1}^{2}+x_{1}\cdot x_{2}}\cdot P-\sigma \leq 0$$

(45) $$g_{3}(x)=\frac{1}{\sqrt{2}\cdot x_{2}+x_{1}}\cdot P-\sigma \leq 0$$

where $0\leq x_{1},x_{2}\leq 1$, $l=100\;\mathrm{cm},P=\sigma =2\;kN/cm^{2}$.

(4) Welded beam design problem

The welded beam design problem is a classic engineering optimization problem widely applied in scenarios such as crane beams, bridge structures, steel frame buildings, and heavy equipment supports. Through optimized design, it is possible to reduce costs, decrease weight, and improve structural strength.

The objective of this problem is to minimize the manufacturing cost of the welded beam by optimizing geometric parameters such as weld thickness ($x_{1}$), beam width ($x_{2}$), beam thickness ($x_{3}$) and weld length ($x_{4}$). At the same time, it must satisfy multiple mechanical performance constraints, including limitations on shear strength, bending load, and buckling stress. Additionally, the weld width and deflection must remain within allowable limits to ensure the stiffness and stability of the beam.

(46) $$\min\;\;f(x)=1.10471x_{1}^{2}x_{2}+0.04811x_{3}x_{4}(14+x_{2})$$

*s.t.*

(47) $$g_{1}(x)=\tau (x)-\tau_{\max}\leq 0$$

(48) $$g_{2}(x)=\sigma (x)-\sigma_{\max}\leq 0$$

(49) $$g_{3}(x)=x_{1}-x_{4}\leq 0$$

(50) $$g_{4}(x)=0.1047\cdot x_{1}^{2}+0.04811\cdot x_{3}\cdot x_{4}\cdot (14+x_{2})-5.0\leq 0$$

(51) $$g_{5}(x)=0.125-x_{1}\leq 0\;$$

(52) $$g_{6}(x)=\delta (x)-\delta_{\max}\leq 0$$

(53) $$g_{7}(x)=P-P_{c}(x)\leq 0$$

where the parameters with fixed values are as follows. P=6000, L=14, $E=30\times 10^{6}$, $G=12\times 10^{6}$, $\tau_{\max}=13,600$, $\sigma_{\max}=30,000$, $\delta_{\max}=0.25$. There are some equation relationships among variables as follows.

$\tau (x)=\sqrt{{\left(\tau^{\prime }\right)}^{2}+2\cdot \tau^{\prime }\cdot \tau^{\prime \prime }\cdot \frac{x_{2}}{2\cdot R}+{\left(\tau^{\prime \prime }\right)}^{2}}$, $\tau^{\prime }=\frac{P}{\sqrt{2}\cdot x_{1}\cdot x_{2}}$, $\tau^{\prime \prime }=\frac{M\cdot R}{J}$, $M=P\cdot \left(L+\frac{x_{2}}{2}\right)$,$R=\sqrt{\frac{x_{2}^{2}}{4}+{\left(\frac{x_{1}+x_{3}}{2}\right)}^{2}}$, $\delta (x)\;\;\;=\frac{4\cdot P\cdot L^{3}}{E\cdot x_{3}^{3}\cdot x_{4}}$, $\sigma (x)\;\;\;=\frac{6\cdot P\cdot L}{x_{3}^{2}\cdot x_{4}}$, $P_{c}(x)=\left(4.013\cdot E\cdot \frac{x_{3}\cdot x_{4}^{3}}{6\cdot L^{2}}\right)$$\cdot \left(1-\frac{x_{3}}{4}\cdot \sqrt{\frac{E}{L\cdot G}}\right)$, $J=2\cdot \left\{\sqrt{2}\cdot x_{1}\cdot x_{2}\cdot \left[\frac{x_{2}^{2}}{12}+{\left(\frac{x_{1}+x_{3}}{2}\right)}^{2}\right]\right\}$. The range of values for decision variables is $0.1\leq x_{1},x_{4}\leq 2$, $0.1\leq x_{2},x_{3}\leq 10$.

(5) Tension/compression spring design problem

The tension/compression spring design problem is a classic engineering optimization problem with wide-ranging applications across various fields. For example, in mechanical equipment, springs are used for vibration control, energy storage, and impact absorption. In the automotive industry, they are an integral part of suspension systems, providing shock absorption and structural support. In the aerospace sector, springs are employed for high-precision damping and load-bearing. In consumer electronics, they are commonly used in buttons, connectors, and mechanical latches.

The objective of this problem is to minimize the weight of the spring by optimizing its geometric parameters while satisfying multiple performance constraints, including spring deflection, shear stress, natural frequency, and outer diameter limitations. The problem involves three design variables: wire diameter ($x_{1}$), mean coil diameter ($x_{2}$), and the number of active coils ($x_{3}$), all of which directly affect the performance and service life of the spring. The mathematical expression is shown as Formulas (54)–(58).

(54) $$\min\;\;f(x)=(x_{3}+2)\cdot x_{2}\cdot x_{1}^{2}$$

*s.t.*

(55) $$g_{1}(x)=1-\frac{x_{2}^{3}\cdot x_{3}}{71,785\cdot x_{1}^{4}}\leq 0\;$$

(56) $$g_{2}(x)=\frac{4\cdot x_{2}^{2}-x_{1}\cdot x_{2}}{12,566\cdot \left(x_{2}\cdot x_{1}^{3}-x_{1}^{4}\right)}+\frac{1}{5108\cdot x_{1}^{2}}-1\leq 0$$

(57) $$g_{3}(x)=1-\frac{140.45\cdot x_{1}}{x_{2}^{2}\cdot x_{3}}\leq 0$$

(58) $$g_{4}(x)=\frac{x_{1}+x_{2}}{1.5}-1\leq 0$$

where $0.05\leq x_{1}\leq 2,\;\;0.25\leq x_{2}\leq 1.3,\;\;2\leq x_{3}\leq 15$.

(6) Pressure vessel design optimization problem

The pressure vessel design problem is widely applied in fields such as chemical engineering, energy, food and pharmaceutical industries, and aerospace. Examples include reactors, gas storage tanks, and steam generators, which are responsible for storing and transporting liquids and gases under high-pressure conditions. The challenge of this problem lies in handling mixed variables and multiple constraints while minimizing the cost and maintaining the performance of the vessel.

The objective is to minimize the manufacturing cost of a cylindrical pressure vessel, including welding, material, and forming costs. The design variables include shell thickness ($x_{1}$), head thickness ($x_{2}$), inner radius ($x_{3}$), and vessel length ($x_{4}$). For the pressure vessel design problem, the shell thickness $x_{1}$ and head thickness $x_{2}$ are integer-multiple variables with a discretization step of 0.0625, which reflects standard manufacturing requirements in practical engineering design. In this study, these two variables are mapped to the nearest feasible discrete values during function evaluation and result reporting, while $x_{3}$ and $x_{4}$ are treated as continuous variables. This treatment ensures that the evaluated solutions strictly satisfy the original mixed-integer design requirements.

The problem is subject to various constraints, including geometric, material, and mechanical performance constraints, to ensure that the pressure vessel achieves sufficient strength and stability at the lowest possible cost.

(59) $$\min\;\;\;f(x)=0.6224\cdot x_{1}\cdot x_{3}\cdot x_{4}+1.7781\cdot x_{2}\cdot x_{3}^{2}+3.1661\cdot x_{1}^{2}\cdot x_{4}+19.84\cdot x_{1}^{2}\cdot x_{3}$$

*s.t.*

(60) $$g_{1}(x)=-x_{1}+0.0193\cdot x_{3}\leq 0\;$$

(61) $$g_{2}(x)=-x_{2}+0.0954\cdot x_{3}\leq 0$$

(62) $$g_{3}(x)=-\pi x_{3}^{2}-\frac{4}{3}\cdot \pi x_{3}^{3}+1,296,000\leq 0$$

(63) $$\;g_{4}(x)=x_{4}-240\leq 0$$

where $0\leq x_{1},x_{2}\leq 100$, $0\leq x_{3},x_{4}\leq 200$.

#### 4.2.2. Results and Discussions of Solving Engineering Problems

**Figure 4:**
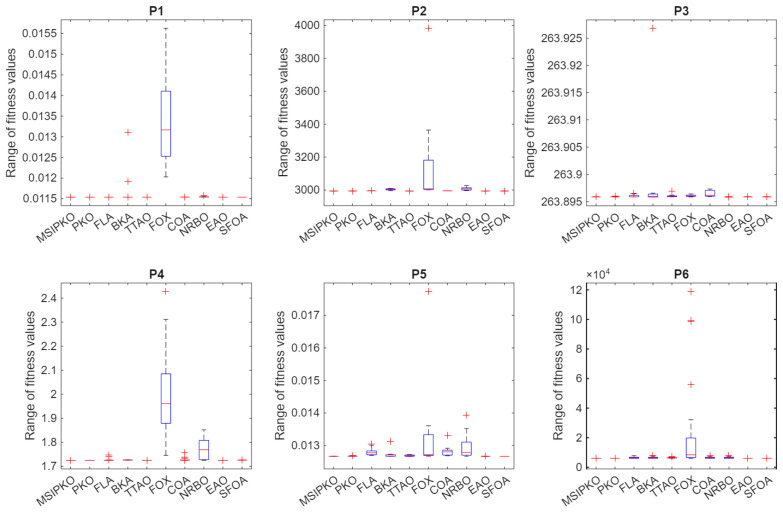
Boxplot of MSIPKO and competitor algorithms for 6 engineering problems.

This section conducts simulation experiments on six representative engineering optimization problems. The numerical simulation conditions and constraint-handling strategies are shown in Table 15. The computational results are summarized in Table 16 and Table 17, where Table 16 presents the results of the first five algorithms and Table 17 lists those of the remaining five algorithms. The distribution of solutions across different algorithms is visualized in Figure 4.

**Figure 5:**
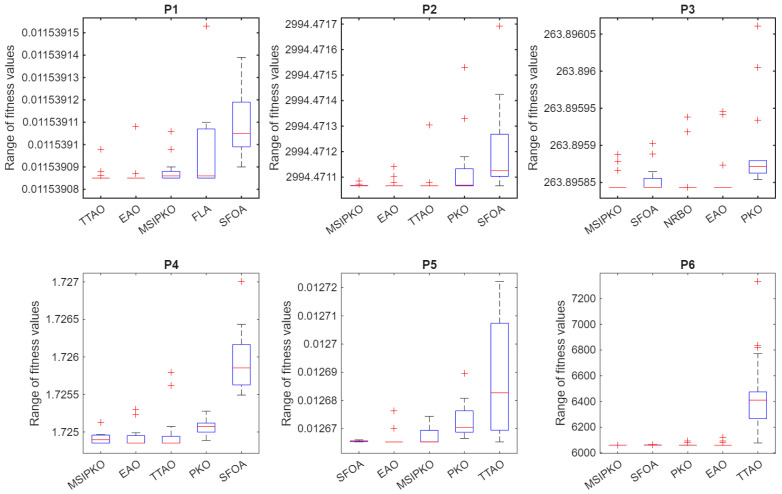
Boxplot of the top five algorithms selected based on mean fitness values for each engineering design problem.

It should be noted that, due to the large dispersion of certain algorithms in some problems, the boxplots in Figure 4 may exhibit compressed regions for other methods. To improve the clarity of visualization and provide a more detailed comparison among the leading algorithms, Figure 5 further presents the boxplots of the top five algorithms selected based on mean fitness values for each problem, where the algorithms are arranged according to their ranking based on mean performance, consistent with the results reported in Table 18, with the leftmost box representing the best-performing algorithm.

The best objective values and their corresponding solution vectors are listed in Table 19. In order to analyze the quality of various algorithms when solving engineering optimization problems, the ranking results based on mean values are shown in Table 18.

For all experiments, a unified experimental setting is adopted to ensure a fair comparison. All algorithms are executed with the same population size and maximum number of iterations, and each problem is independently run 30 times. For problems involving discrete variables, the corresponding constraints are handled within the objective evaluation to ensure feasibility of the obtained solutions.

For problems involving discrete variables, namely the speed reducer design problem (P2) and the pressure vessel design problem (P6), the corresponding integer or discrete constraints are explicitly handled within the objective function evaluation. This ensures that all candidate solutions satisfy the problem-specific feasibility requirements throughout the optimization process. For constrained optimization problems, the original constraint-handling mechanisms of each algorithm are retained, ensuring that the comparison reflects their intrinsic performance under standard configurations.

Based on the ranking results presented in Table 18, a clear overall performance pattern can be observed. MSIPKO achieves the best overall ranking with the lowest total rank score, indicating its strong competitiveness across different types of engineering optimization problems. Specifically, MSIPKO ranks first in four out of six problems, while maintaining competitive performance in the remaining two cases. Compared with other algorithms, such as PKO and EAO, which perform well on certain individual problems, MSIPKO demonstrates a more balanced and consistent performance across all test cases. In contrast, algorithms such as FLA and FOX obtain relatively poor rankings, reflecting their limited adaptability to complex constrained optimization scenarios.

A more detailed analysis of each problem is given as follows.

For the I-beam deflection problem (P1), MSIPKO ranks third in terms of mean performance. Although it does not achieve the best result, less than TTAO and EAO, its solution is still very close to the theoretical optimum. Meanwhile, the distribution shown in Figure 4 indicates that MSIPKO maintains relatively stable convergence with limited fluctuations. Compared with some algorithms that exhibit larger deviations, MSIPKO provides a more reliable performance.

For the speed reducer design problem (P2), MSIPKO achieves the best overall performance. The obtained mean value is highly competitive, and the variance is relatively small, indicating both high accuracy and stability. Compared with algorithms such as FLA and FOX, which show larger deviations and fluctuations, MSIPKO demonstrates stronger adaptability to nonlinear and mixed-variable constraints.

For the three-bar truss structure problem (P3), most algorithms obtain results very close to the theoretical optimum. MSIPKO achieves the best mean performance, although the performance gap among the top algorithms is relatively small. From Figure 4 and Figure 5, it can be observed that MSIPKO exhibits relatively stable distributions with fewer outliers, indicating consistent convergence behavior.

For the welded beam design problem (P4), MSIPKO ranks first and achieves the best mean performance among all algorithms. Compared with recent algorithms such as COA and TTAO, MSIPKO shows better convergence accuracy and lower variance, demonstrating its effectiveness in handling nonlinear engineering constraints.

For the spring design problem (P5), MSIPKO ranks third, slightly behind the top-performing algorithms (including EAO and SFOA). However, the difference in objective value is extremely small, indicating that MSIPKO still maintains a competitive performance. In addition, its solution distribution remains relatively concentrated, suggesting stable convergence across multiple runs.

Finally, in the pressure vessel design problem (P6), MSIPKO achieves the best performance with a mean value of 6059.976678 and a very low standard deviation of 0.419671. This indicates that the proposed algorithm maintains both high solution quality and strong stability under the mixed discrete–continuous design constraints. In contrast, algorithms such as FOX, FLA and BKA exhibit much larger fluctuations, suggesting relatively weaker robustness in this problem. For the pressure vessel problem, the reported values of $x_{1}$ and $x_{2}$ are the discrete feasible values obtained after applying the 0.0625-step mapping in Table 19.

### 4.3. Discussion

Based on the comprehensive experimental results presented in Section 4.1 and Section 4.2, the proposed MSIPKO demonstrates competitive and generally superior performance across both benchmark functions and engineering optimization problems.

In Section 4.1, MSIPKO is evaluated on 12 constrained functions from the CEC 2006 test suite. The results show that the proposed algorithm achieves the best or near-best performance on most functions in terms of mean value, best value, and robustness. The convergence analysis further indicates that MSIPKO exhibits efficient search behavior across different evaluation budgets, maintaining a good balance between global exploration and local exploitation. In addition, the ablation study confirms that each component (C1, C2, and C3) contributes positively to the overall performance, and their integration leads to further improvements.

Despite these promising results, several limitations should be acknowledged. First, the current evaluation is mainly conducted on medium-scale constrained optimization problems (CEC 2006), and the performance of MSIPKO on high-dimensional or large-scale problems has not yet been fully investigated. Second, the introduction of additional modules leads to a slight increase in per-iteration computational overhead, which may affect efficiency in scenarios with strict real-time requirements. Third, the constraint-handling strategy adopted in this study is relatively standard, and its effectiveness in more complex or dynamic constraint environments still requires further validation.

Beyond these limitations, it is also important to consider the applicability of MSIPKO to more complex and emerging constrained optimization scenarios. In recent studies, constrained optimization problems have been formulated in increasingly complex forms, such as energy system scheduling under operational constraints and security-oriented optimization problems with implicit or hidden constraints. These problems are often characterized by highly irregular feasible regions, strong coupling among variables, and complex constraint structures.

Although MSIPKO demonstrates strong performance in the tested problems, its applicability to such emerging scenarios has not yet been fully investigated. Nevertheless, due to its enhanced exploration capability and adaptability, MSIPKO has the potential to be extended to these complex optimization tasks, which represents a promising direction for future research.

## 5. Conclusions and Future Work

This paper proposes a multi-strategy improved pied kingfisher optimizer (MSIPKO) to enhance the performance of the original PKO for constrained optimization problems. By integrating a reverse differential crossover mechanism, an enhanced diving-fishing operator, and a refined commensalism phase, the proposed algorithm improves global exploration, local exploitation, and convergence stability without changing the asymptotic computational complexity.

Comprehensive experiments on 12 constrained benchmark functions from the CEC 2006 test suite and six classical engineering design problems demonstrate that MSIPKO achieves competitive and generally superior performance compared with several state-of-the-art algorithms. In particular, the results indicate improvements in solution quality, robustness, and convergence behavior across most test cases.

Additional analyses, including ablation studies and sensitivity evaluations under different function evaluation budgets, further verify the effectiveness of the proposed components and the stability of the algorithm. Moreover, comparisons with different constraint-handling strategies confirm the robustness of MSIPKO across diverse constraint environments.

Despite these promising results, several limitations remain, as discussed in Section 4.3, and addressing them will be an important direction for future work.

In future research, we will focus on extending MSIPKO to high-dimensional and large-scale constrained optimization problems, improving its computational efficiency through parallelization, and enhancing its adaptability by integrating more advanced constraint-handling mechanisms. Furthermore, extending the proposed method to multi-objective optimization problems and real-world applications, such as energy scheduling and UAV path planning, will also be investigated.

## Figures and Tables

**Table 1:** Summary of representative methods for constrained optimization problems.

Category	Representative Methods	Key Advantages	Limitations
Constraint-handling methods	Penalty method [2], Deb’s rules [3], multi-objective transformation [4], ε-constraint [5], dual-population strategies [6]	Effective feasibility handling; simple or flexible implementation	Sensitive to parameters; may introduce additional computational complexity
Swarm intelligence algorithms	HFOA [9], BCO [10], DBO [11], PLO [12], SMOA [13]	Strong global exploration capability; easy to implement	Difficulty in maintaining population diversity; imbalance between exploration and exploitation
Hybrid/Improved algorithms	EAOA [14], FILPSO-SCAε [15], AMaOTCO [16], SHGWJA [17]	Improved convergence accuracy and solution quality	Increased algorithmic complexity; reduced generality due to multiple strategies
Engineering applications	ACS-CDE [23], ISSA [24], MACSGWO [26], IDBO [29], NBESOA [30]	Effective in solving real-world constrained problems	Strong problem dependence; limited generalization ability

**Table 2:** Comparison of computational complexity of PKO and MSIPKO modules.

Algorithms	PKO	MSIPKO
Initialization	$O\left(N\cdot D\right)$	$O\left(N\cdot D\right)$
Implement reverse differential crossover mechanism	—	$O\left(N\cdot D\right)$
Hovering/perching	$O\left(N\cdot D\right)$	$O\left(N\cdot D\right)$
Diving	$O\left(N\cdot D\right)$	$O\left(N\cdot D\right)$
Commensalism	$O\left(N\cdot D\right)$	$O\left(N\cdot D\right)$
Complexity of per-iteration	$O\left(3\cdot N\cdot D\right)$	$O\left(4\cdot N\cdot D\right)$
Total	$O\left[\left(3\cdot T+1\right)\cdot N\cdot D\right]$	$O\left[\left(4\cdot T+1\right)\cdot N\cdot D\right]$

**Table 3:** The characteristics of CEC 2006 benchmark functions (G01–G12).

Function	n	Type of Function	ρ	Optimal	LI	NI	LE	NE	a
G01	13	Quadratic	0.0111%	−15	9	0	0	0	6
G02	20	Non-linear	99.9971%	−0.8036019	0	2	0	0	1
G03	10	Polynomial	0.0000%	−1.0005	0	0	0	1	1
G04	5	Quadratic	52.1230%	−30,665.5387	0	6	0	0	2
G05	4	Cubic	0.0000%	5126.4967	2	0	0	3	3
G06	2	Cubic	0.0066%	−6961.8139	0	2	0	0	2
G07	10	Quadratic	0.0003%	24.3062	3	5	0	0	6
G08	2	Non-linear	0.8560%	−0.095825	0	2	0	0	0
G09	7	Polynomial	0.5121%	680.630057	0	4	0	0	2
G10	8	Linear	0.0010%	7049.2480	3	3	0	0	6
G11	2	Quadratic	0.0000%	0.7499	0	0	0	1	1
G12	3	Quadratic	4.7713%	−1	0	1	0	0	0

**Table 4:** Numerical simulation conditions of 10 algorithms for solving the CEC 2006 benchmark functions.

Function	Population Size	Iterations	FEs	Constraint-Handling Strategies
G01	100	500	50,000	Static penalty functions
G02	100	2000	200,000	Static penalty functions
G03	100	2000	200,000	Static penalty functions
G04	100	200	20,000	Static penalty functions
G05	100	500	50,000	Static penalty functions
G06	100	100	10,000	Static penalty functions
G07	100	2000	200,000	Static penalty functions
G08	50	20	1000	Static penalty functions
G09	100	500	50,000	Static penalty functions
G10	100	2000	200,000	Static penalty functions
G11	100	200	20,000	Static penalty functions
G12	40	50	2000	Static penalty functions

**Table 5:** Comparison results of MSIPKO and nine other algorithms for 12 benchmark functions (1).

Functions	Statistics	MSIPKO	PKO	FLA	BKA	TTAO
G01	Mean	**−15**	−12.9328	−10.6969	−4.6878	−15.0000
	SD	**0**	1.5953	3.2672	1.7670	1.0739 × 10^−8^
	Best	**−15**	**−15**	**−15**	−9.6339	**−15**
	Worst	**−15**	−9	−5	−2	−15.0000
G02	Mean	**−0.801095**	−0.784224	−0.584017	−0.605223	−0.622355
	SD	**4.5681 × 10^−3^**	1.6510 × 10^−2^	9.2062 × 10^−2^	9.1242 × 10^−2^	1.1827 × 10^−1^
	Best	**−0.803619**	−0.803489	−0.777835	−0.750609	−0.792564
	Worst	**−0.787683**	−0.752728	−0.434697	−0.330256	−0.482724
G03	Mean	**−1.000499**	−0.260171	−0.899194	−0.953200	−0.900470
	SD	**1.0675 × 10^−6^**	2.1712 × 10^−1^	2.3262 × 10^−1^	1.4425 × 10^−1^	5.3299 × 10^−2^
	Best	**−1.000500**	−0.869920	−0.999118	−1.000481	−0.969864
	Worst	**−1.000497**	−0.045100	−0.132929	−0.482825	−0.781659
G04	Mean	**−30,665.5387**	−30,664.4202	−30,643.9203	−30,654.0000	−30,665.5382
	SD	**1.6229 × 10^−7^**	9.4643 × 10^−1^	9.1300 × 10^1^	6.1857 × 10^1^	4.5989 × 10^−4^
	Best	**−30,665.5387**	−30,665.4131	−30,665.5292	**−30,665.5387**	**−30,665.5387**
	Worst	**−30,665.5387**	−30,661.8847	−30,164.6536	−30,326.5193	−30,665.5369
G05	Mean	**5126.4967**	5131.8063	5297.8303	5167.6899	5172.7790
	SD	**3.4771 × 10^−6^**	4.1160 × 10^0^	6.7773 × 10^2^	1.1289 × 10^2^	2.6924 × 10^1^
	Best	**5126.4967**	5126.5416	5129.2076	5126.5046	5134.1553
	Worst	**5126.4967**	5146.5100	8880.0000	5668.8127	5232.5777
G06	Mean	**−6961.8139**	−6911.6279	−6950.9430	−6961.8033	−6942.0068
	SD	**4.6252 × 10^−12^**	4.5712 × 10^1^	2.0330 × 10^1^	3.6077 × 10^−2^	1.9922 × 10^1^
	Best	**−6961.8139**	−6959.7614	−6961.8028	**−6961.8139**	−6960.9252
	Worst	**−6961.8139**	−6754.1389	−6864.8359	−6961.6417	−6855.1227
G07	Mean	**24.3198**	25.0260	27.5585	64.5204	24.8721
	SD	**1.7424 × 10^−2^**	3.7751 × 10^−1^	7.6560 × 10^0^	6.4747 × 10^1^	3.8262 × 10^−1^
	Best	**24.3072**	24.5466	24.6259	24.9487	24.3303
	Worst	**24.4005**	25.9218	66.1651	255.2172	25.6463
G08	Mean	**−0.095825**	−0.087289	−0.089219	−0.095631	−0.090976
	SD	**4.7744 × 10^−8^**	1.4150 × 10^−2^	1.6868 × 10^−2^	6.5436 × 10^−4^	1.5199 × 10^−2^
	Best	**−0.095825**	−0.095773	**−0.095825**	**−0.095825**	**−0.095825**
	Worst	**−0.095825**	−0.028362	−0.021295	−0.092850	−0.02 8383
G09	Mean	**680.630069**	680.7278	680.6743	686.0756	680.6838
	SD	**1.4812 × 10^−5^**	6.8481 × 10^−2^	3.8327 × 10^−2^	1.2373 × 10^1^	3.3064 × 10^−2^
	Best	**680.630058**	680.6538	680.6352	680.6658	680.6444
	Worst	**680.630109**	680.9750	680.8504	749.9112	680.8121
G10	Mean	**7049.2556**	7477.0739	8007.5129	8062.1063	7381.5745
	SD	**1.3602 × 10^−2^**	1.6362 × 10^2^	2.1611 × 10^3^	1.3527 × 10^3^	1.0448 × 10^2^
	Best	**7049.2480**	7248.9473	7101.2679	7100.9835	7127.7024
	Worst	**7049.2985**	7837.7985	19262.2455	14183.4060	7507.3094
G11	Mean	**0.749900**	0.749917	0.749943	0.749900	0.749922
	SD	**1.1292 × 10^−16^**	1.1002 × 10^−5^	1.8628 × 10^−4^	4.5718 × 10^−8^	3.0927 × 10^−5^
	Best	**0.749900**	0.749902	**0.749900**	**0.749900**	**0.749900**
	Worst	**0.749900**	0.749943	0.750918	**0.749900**	0.750063
G12	Mean	**−1**	−0.9885	−0.9808	−0.9969	−0.9963
	SD	**0**	9.4945 × 10^−3^	4.9019 × 10^−2^	4.2583 × 10^−3^	4.1849 × 10^−3^
	Best	**−1**	−0.999857	−0.999996	−1.000000	−0.999963
	Worst	**−1**	−0.959461	−0.748302	−0.986384	−0.988343

**Table 6:** Comparison results of MSIPKO and nine other algorithms for 12 benchmark functions (2).

Functions	Statistics	FOX	COA	NRBO	EAO	SFOA
G01	Mean	−14.752	−7.7689	−7.9520	−1.4333	−3.0620
	SD	7.2542 × 10^−1^	2.4114	2.7684	2.1764 × 10^0^	2.2961 × 10^0^
	Best	−14.9970	−12.9997	−14.5628	−9.0000	−6.1204
	Worst	−12.0019	−5	−4	0	0.9308
G02	Mean	−0.378660	−0.520760	−0.450921	−0.643042	−0.649821
	SD	9.6609 × 10^−2^	7.1374 × 10^−2^	5.4564 × 10^−2^	4.3071 × 10^−2^	3.9996 × 10^−2^
	Best	−0.631265	−0.633044	−0.564679	−0.769960	−0.760860
	Worst	−0.242575	−0.360306	−0.409150	−0.544209	−0.604702
G03	Mean	0.189077	−0.479319	−0.611842	−0.784701	−1.000494
	SD	1.5044 × 10^−1^	2.5810 × 10^−1^	2.3377 × 10^−1^	1.2331 × 10^−1^	3.9296 × 10^−6^
	Best	0.000264	−0.971089	−0.993403	−0.997542	−1.000500
	Worst	0.605330	−0.087333	−0.194243	−0.581804	−1.000487
G04	Mean	−30,665.5252	−30,659.4466	−30,647.7918	−30,665.5387	−29,370.2824
	SD	1.9102 × 10^−2^	3.5737 × 10^0^	3.3011 × 10^1^	0.0000	258.7623
	Best	−30,665.5383	−30,663.9331	**−30,665.5387**	−30,665.5387	−29,821.7131
	Worst	−30,665.4357	−30,649.9932	−30,505.8409	−30,665.5387	−28,923.8142
G05	Mean	5895.9748	5174.2454	5163.6804	5126.4967	5127.1523
	SD	4.3731 × 10^2^	3.2125 × 10^1^	4.3675 × 10^1^	9.2504 × 10^−13^	3.8612 × 10^−1^
	Best	5217.8893	5131.1896	5126.5740	5126.4967	5126.6045
	Worst	7138.3198	5255.6888	5320.2518	5126.4967	5128.2331
G06	Mean	−6961.8093	−6861.8147	−6961.7942	47,315.0427	−6948.2226
	SD	3.6448 × 10^−3^	1.0728 × 10^2^	6.0161 × 10^−2^	5.2476 × 10^4^	1.2603 × 10^1^
	Best	−6961.8136	−6948.3252	−6961.8137	−6961.8139	−6960.8697
	Worst	−6961.8007	−6436.4211	−6961.4908	102,027	−6906.5417
G07	Mean	24.6501	24.6876	51.5447	24.3062	24.3399
	SD	2.2702 × 10^−1^	2.0969 × 10^−1^	1.5166 × 10^1^	3.3741 × 10^−10^	1.5397 × 10^−2^
	Best	24.3697	24.3890	33.6358	24.3062	24.3149
	Worst	25.4220	25.1874	103.1055	24.3062	24.3928
G08	Mean	−0.084489	−0.073303	−0.095753	−0.095825	−0.086815
	SD	2.5789 × 10^−2^	2.7167 × 10^−2^	3.8616 × 10^−4^	7.0748 × 10^−7^	8.7847 × 10^−3^
	Best	**−0.095825**	−0.095823	**−0.095825**	−0.095825	−0.094781
	Worst	−0.025812	−0.018921	−0.093709	−0.095823	−0.066377
G09	Mean	680.6878	680.7354	688.8294	680.630057	680.7385
	SD	6.8670 × 10^−2^	5.8676 × 10^−2^	5.0177 × 10^0^	5.7815 × 10^−13^	4.0766 × 10^−2^
	Best	680.6329	680.6516	681.2589	680.630057	680.6628
	Worst	680.9834	680.8638	699.2373	680.630057	680.8253
G10	Mean	15,982.4803	7525.6372	9692.0477	7049.2480	7074.2383
	SD	3.3113 × 10^3^	2.4202 × 10^2^	1.2261 × 10^3^	2.3256 × 10^−7^	3.6626 × 10^1^
	Best	10,408.6164	7116.1744	7838.2872	7049.2480	7054.3972
	Worst	24,921.1137	8303.2140	12,886.3826	7049.2480	7251.9313
G11	Mean	0.749900	0.750092	0.749900	0.749900	0.749942
	SD	3.2616 × 10^−8^	1.8700 × 10^−4^	2.4650 × 10^−8^	2.2775 × 10^−12^	6.7297 × 10^−5^
	Best	**0.749900**	0.749904	**0.749900**	0.749900	0.749900
	Worst	**0.749900**	0.750653	**0.749900**	0.749900	0.750134
G12	Mean	−0.9508	−0.9908	−0.9971	−0.9999	−0.9935
	SD	4.4286 × 10^−2^	1.2031 × 10^−2^	3.8968 × 10^−3^	6.0000 × 10^−7^	2.7150 × 10^−3^
	Best	−1.0000	−1.0000	−1.0000	−0.9999	−0.9996
	Worst	−0.8500	−0.9547	−0.9833	−0.9999	−0.9877

**Table 7:** Ranking results of the 10 algorithms by the mean value and standard deviation.

	MSIPKO	PKO	FLA	BKA	TTAO	FOX	COA	NRBO	EAO	SFOA
G01	1	4	5	8	2	3	7	6	10	9
G02	1	2	7	6	5	10	8	9	4	3
G03	1	9	5	3	4	10	8	7	6	2
G04	2	5	9	7	3	4	6	8	1	10
G05	2	4	9	6	7	10	8	5	1	3
G06	1	8	5	3	7	2	9	4	10	6
G07	2	7	8	10	6	4	5	9	1	3
G08	1	7	6	4	5	9	10	3	2	8
G09	2	6	3	9	4	5	7	10	1	8
G10	2	5	7	8	4	10	6	9	1	3
G11	1	6	9	1	7	1	10	1	1	8
G12	1	8	9	4	5	10	7	3	2	6
Total rank	**17**	71	82	69	59	78	91	74	40	69
Mean rank	**1.42**	5.92	6.83	5.75	4.92	6.5	7.58	6.17	3.33	5.75
Final rank	**1**	6	9	4	3	8	10	7	2	4

**Table 8:** Results of the Wilcoxon test including Bonferroni correction between MSIPKO and other algorithms.

MSIPKO vs.	R+	R−	*p*-Value	Bonferroni-Corrected *p*-Value	α=0.05	α=0.1	α=0.2
**PKO**	**78**	0	0.0005	0.0045	H1	H1	H1
**FLA**	**78**	0	0.0005	0.0045	H1	H1	H1
**BKA**	**78**	0	0.0005	0.0045	H1	H1	H1
**TTAO**	**78**	0	0.0005	0.0045	H1	H1	H1
**FOX**	**78**	0	0.0005	0.0045	H1	H1	H1
**COA**	**78**	0	0.0005	0.0045	H1	H1	H1
**NRBO**	**78**	0	0.0005	0.0045	H1	H1	H1
**EAO**	**53**	25	0.0312	0.2808	H0	H0	H0
**SFOA**	**78**	0	0.0005	0.0045	H1	H1	H1

**Table 9:** Comparison results of MSIPKO’s component-wise ablation study for 12 benchmark functions.

Functions	Statistics	MSIPKO	PKO	PKO + C1	PKO + C2	PKO + C3
G01	Mean	−15.0000	−12.9328	−15.0000	−13.8151	−13.0992
	SD	0.0000 × 10^0^	1.5953 × 10^0^	0.0000 × 10^0^	1.6750 × 10^0^	2.5502 × 10^0^
	Best	−15.0000	−15.0000	−15.0000	−15.0000	−15.0000
	Worst	−15.0000	−9.0000	−15.0000	−9.0000	−6.0000
G02	Mean	−0.801095	−0.784224	−0.801510	−0.787246	−0.793628
	SD	4.5681 × 10^−3^	1.6510 × 10^−2^	3.0839 × 10^−3^	1.2168 × 10^−2^	5.6274 × 10^−3^
	Best	−0.803619	−0.803489	−0.803567	−0.802704	−0.802903
	Worst	−0.787683	−0.752728	−0.794606	−0.763718	−0.784999
G03	Mean	−1.000499	−0.260171	−0.990002	−1.000499	−0.999379
	SD	1.0675 × 10^−6^	2.1712 × 10^−1^	3.7131 × 10^−3^	5.2850 × 10^−6^	6.9107 × 10^−4^
	Best	−1.000500	−0.869920	−1.000419	−1.000500	−1.000199
	Worst	−1.000497	−0.045100	−0.980334	−1.000497	−0.997557
G04	Mean	−30,665.5387	−30,664.4202	−30,665.4029	−30,665.5387	−30,664.7662
	SD	1.6229 × 10^−7^	9.4643 × 10^−1^	1.3348 × 10^−1^	3.0327 × 10^−6^	5.7969 × 10^−1^
	Best	−30,665.5387	−30,665.4131	−30,665.5338	−30,665.5387	−30,665.3512
	Worst	−30,665.5387	−30,661.8847	−30,664.9830	−30,665.5387	−30,663.5512
G05	Mean	5126.4967	5131.8063	5128.6840	5126.4967	5129.8834
	SD	3.4771 × 10^−6^	4.1160 × 10^0^	1.4021 × 10^0^	4.8691 × 10^−5^	1.4218 × 10^0^
	Best	5126.4967	5126.5416	5126.5351	5126.4967	5128.2228
	Worst	5126.4967	5146.5100	5132.1536	5126.4970	5133.0762
G06	Mean	−6961.8139	−6911.6279	−6961.7910	−6961.8084	−6928.3668
	SD	4.6252 × 10^−12^	4.5712 × 10^1^	3.7083 × 10^−2^	4.5509 × 10^−3^	2.0436 × 10^1^
	Best	−6961.8139	−6959.7614	−6961.8139	−6961.8136	−6953.5894
	Worst	−6961.8139	−6754.1389	−6961.6505	−6961.7974	−6894.9480
G07	Mean	24.3198	25.0260	24.3751	24.3063	24.4275
	SD	1.7424 × 10^−2^	3.7751 × 10^−1^	3.3608 × 10^−2^	7.2269 × 10^−15^	5.9871 × 10^−2^
	Best	24.3072	24.5466	24.3227	24.3063	24.3462
	Worst	24.4005	25.9218	24.4719	24.3063	24.5663
G08	Mean	−0.095825	−0.087289	−0.095811	−0.095793	−0.093862
	SD	4.7744 × 10^−8^	1.4150 × 10^−2^	1.1623 × 10^−5^	4.9728 × 10^−5^	2.0669 × 10^−3^
	Best	−0.095825	−0.095773	−0.095825	−0.095825	−0.095691
	Worst	−0.095825	−0.028362	−0.095779	−0.095619	−0.087634
G09	Mean	680.630069	680.727834	680.693395	680.630147	680.716837
	SD	1.4812 × 10^−5^	6.8481 × 10^−2^	3.5785 × 10^−2^	1.8069 × 10^−4^	4.5802 × 10^−2^
	Best	680.630058	680.653825	680.639797	680.630063	680.671101
	Worst	680.630109	680.974952	680.762383	680.630612	680.805783
G10	Mean	7049.2556	7477.0739	7225.0307	7049.7022	7323.9559
	SD	1.3602 × 10^−2^	1.6362 × 10^2^	9.8351 × 10^1^	1.5699 × 10^0^	1.0776 × 10^2^
	Best	7049.2480	7248.9473	7057.5817	7049.2482	7087.7342
	Worst	7049.2985	7837.7985	7491.3307	7055.4735	7511.1648
G11	Mean	0.749900	0.749917	0.749915	0.749900	0.753134
	SD	1.1292 × 10^−16^	1.1002 × 10^−5^	2.5707 × 10^−5^	1.9352 × 10^−13^	2.7618 × 10^−3^
	Best	0.749900	0.749902	0.749900	0.749900	0.750276
	Worst	0.749900	0.749943	0.749982	0.749900	0.759891
G12	Mean	−1.0000	−0.9885	−1.0000	−1.0000	−1.0000
	SD	0.0000 × 10^0^	9.4945 × 10^−3^	0.0000 × 10^0^	5.9989 × 10^−6^	1.1720 × 10^−5^
	Best	−1.000000	−0.999857	−1.000000	−1.000000	−0.999999
	Worst	−1.000000	−0.959461	−1.000000	−0.999975	−0.999970

**Table 10:** Ranking results of the component-wise ablation by the mean value and standard deviation.

	MSIPKO	PKO	PKO + C1	PKO + C2	PKO + C3
G01	1	4	1	3	5
G02	2	5	1	4	3
G03	1	5	4	2	3
G04	1	5	3	2	4
G05	1	5	3	2	4
G06	1	5	3	2	4
G07	2	5	3	1	4
G08	1	5	2	3	4
G09	1	5	3	2	4
G10	1	5	3	2	4
G11	1	4	3	2	5
G12	1	5	1	3	4
Total rank	14	58	30	28	48
Mean rank	1.167	4.83	2.5	2.33	4.00
Final rank	1	5	3	2	4

**Table 11:** Function evaluation budgets and constraint-handling strategies of the compared algorithms on the 12 CEC 2006 constrained benchmark functions (G01–G12).

Function	MSIPKO	HMICA	BSA-SAε	SMA-GM	AGWO	IChoA
**G01**	50,000	200,000	350,000	30,000	30,000	30,000
**G02**	200,000	200,000	350,000	30,000	30,000	30,000
**G03**	200,000	200,000	350,000	30,000	30,000	30,000
**G04**	20,000	20,000	350,000	30,000	30,000	30,000
**G05**	50,000	200,000	350,000	30,000	30,000	30,000
**G06**	10,000	80,000	350,000	30,000	30,000	30,000
**G07**	200,000	200,000	350,000	30,000	30,000	30,000
**G08**	1000	1000	350,000	30,000	30,000	30,000
**G09**	50,000	200,000	350,000	30,000	30,000	30,000
**G10**	200,000	200,000	350,000	30,000	30,000	30,000
**G11**	20,000	25,000	350,000	30,000	30,000	30,000
**G12**	2000	2000	350,000	30,000	30,000	30,000
**Constraints handling strategies**	Static penalty functions	Deb’s rules	ε-constrained method	Dynamic penalty	Dynamic penalty	Dynamic penalty

**Table 12:** Comparison results of MSIPKO and five other algorithms using different constraint-handling strategies on 12 benchmark functions.

Functions	Statistics	MSIPKO	HMICA	BSA-SAε	SMA-GM	AGWO	IChoA
G01	Mean	**−15**	**−15**	**−15**	−14.834	−7.8403	−12.915
	SD	**0**	**0**	**0**	0.0591	1.7856	1.5116
	Best	**−15**	**−15**	**−15**	−15	−11.854	−14.954
	Worst	**−15**	**−15**	**−15**	−14.692	−5	−1.5116
G02	Mean	**−0.801095**	−0.7942587	−0.791922	−0.5378	−0.5622	−0.775
	SD	**4.5681 × 10^−3^**	0.006486	5.48 × 10^−3^	0.1125	0.0535	0.0143
	Best	**−0.803619**	**−0.803619**	−0.803599	−0.7779	−0.7127	−0.7916
	Worst	**−0.787683**	−0.7826	−0.77988	−0.3011	0.0535	−0.733
G03	Mean	**−1.000499**	−0.992825	−1.000486	−1	−0.9658	−0.9936
	SD	**1.0675 × 10^−6^**	0.0108249	1.64 × 10^−5^	2.51 × 10^−8^	0.0123	0.0019
	Best	**−1.000500**	−1.0004	−1.000498	−1	−0.9863	−0.9963
	Worst	**−1.000497**	−0.95767	−1.000419	−1	−0.9363	−0.9897
G04	Mean	**−30,665.5387**	**−30,665.539**	**−30,665.5 4**	**−30,666**	−30,652	−30,664
	SD	1.6229 × 10^−7^	**0**	**0**	1.7 × 10^−3^	7.8038	1.1844
	Best	**−30,665.5387**	**−30,665.539**	**−30,665.5 4**	**−30,666**	−30,663	−30,665
	Worst	**−30,665.5387**	**−30,665.539**	**−30,665.5 4**	−30,665	−30,628	−30,663
G05	Mean	**5126.4967**	5127.8809	**5126.497**	5239.8	5263.2	5161.4
	SD	**3.4771 × 10^−6^**	1.75067	**0**	96.915	48.62	11.303
	Best	**5126.4967**	5126.497	**5126.497**	5126.5	5156.5	5140.1
	Worst	**5126.4967**	5133.3178	**5126.497**	5466.4	5311.4	5186.9
G06	Mean	**−6961.8139**	**−6961.814**	**−6961.814**	**−6961.8**	1.4141 × 10^18^	−6958.8
	SD	4.6252 × 10^−12^	**0**	**0**	0.0236	7.7453 × 10^18^	2.2065
	Best	**−6961.8139**	**−6961.814**	**−6961.814**	**−6961.8**	−6950.4	−6960.7
	Worst	**−6961.8139**	**−6961.814**	**−6961.814**	−6961.7	4.24 × 10^19^	−6949.8
G07	Mean	**24.3198**	24.4886	24. 3463	25.207	32.642	26.184
	SD	**1.7424 × 10^−2^**	0.1743	4.05 × 10^−2^	0.4071	396.15	0.4666
	Best	24.3072	24.3068	**24.3061**	24.38	32.642	25.374
	Worst	**24.4005**	24.8539	24.5316	27.376	969	27.474
G08	Mean	**−0.095825**	**−0.095825**	**−0.095825**	−0.0846	**−0.0958**	**−0.0958**
	SD	4.7744 × 10^−8^	**0**	**0**	0.0256	2.21 × 10^−6^	1.61 × 10^−17^
	Best	**−0.095825**	**−0.095825**	**−0.095825**	−0.0954	**−0.0958**	**−0.0958**
	Worst	**−0.095825**	**−0.095825**	**−0.095825**	−0.0255	**−0.0958**	**−0.0958**
G09	Mean	**680.630069**	680.6329	680.6302	680.8	712.36	680.88
	SD	**1.4812 × 10^−5^**	0.001461	1.95 × 10^−4^	0.1062	48.318	0.0755
	Best	**680.630058**	680.6308	680.6301	680.65	684.36	680.76
	Worst	**680.630109**	680.6357	680.6310	681.12	901.86	681.13
G10	Mean	**7049.2556**	7283.9543	7053.177	7844.4	8489.1	8197.3
	SD	**1.3602 × 10^−2^**	117.9793	6.04 × 10^0^	349.86	358.25	395.94
	Best	**7049.2480**	7050.5895	7049.278	7065.2	7774.5	7651.4
	Worst	**7049.2985**	7469.1997	7071.253	8546.4	9092.6	8686.3
G11	Mean	**0.749900**	**0.7499**	**0.7499**	**0.75**	0.7501	**0.75**
	SD	1.1292 × 10^−16^	**0**	**0**	1.77 × 10^−5^	8.05 × 10^−5^	1.09 × 10^−5^
	Best	**0.749900**	**0.7499**	**0.7499**	**0.75**	0.7501	**0.75**
	Worst	**0.749900**	**0.7499**	**0.7499**	0.7501	0.7503	0.7501
G12	Mean	**−1**	**−1**	**−1**	**−1**	**−1**	**−1**
	SD	**0**	**0**	**0**	**0**	1.69 × 10^−7^	**0**
	Best	**−1**	**−1**	**−1**	**−1**	**−1**	**−1**
	Worst	**−1**	**−1**	**−1**	**−1**	**−1**	**−1**

**Table 13:** Comparison results of the MSIPKO and three dynamic penalty function-based algorithms under the same simulation settings.

Functions	Statistics	MSIPKO	SMA-GM	AGWO	IChoA
G01	Mean	−14.8000	**−14.834**	−7.8403	−12.915
	SD	7.6112 × 10^−1^	**0.0591**	1.7856	1.5116
	Best	**−15.0000**	**−15**	−11.854	−14.954
	Worst	−12.0000	**−14.692**	−5	−1.5116
G02	Mean	**−0.795288**	−0.5378	−0.5622	−0.775
	SD	**7.1825 × 10^−3^**	0.1125	0.0535	0.0143
	Best	**−0.803531**	−0.7779	−0.7127	−0.7916
	Worst	**−0.777743**	−0.3011	0.0535	−0.733
G03	Mean	−0.900114	**−1**	−0.9658	−0.9936
	SD	6.8601 × 10^−4^	**2.51 × 10^−8^**	0.0123	0.0019
	Best	−0.900461	**−1**	−0.9863	−0.9963
	Worst	−0.896726	**−1**	−0.9363	−0.9897
G05	Mean	**5126.4967**	5239.8	5263.2	5161.4
	SD	**4.6252 × 10^−12^**	96.915	48.62	11.303
	Best	**5126.4967**	5126.5	5156.5	5140.1
	Worst	**5126.4967**	5466.4	5311.4	5186.9
G07	Mean	**24.3752**	25.207	32.642	26.184
	SD	**5.9393 × 10^−2^**	0.4071	396.15	0.4666
	Best	**24.312329**	24.38	32.642	25.374
	Worst	**24.574807**	27.376	969	27.474
G09	Mean	**680.630375**	680.8	712.36	680.88
	SD	**5.1841 × 10^−4^**	0.1062	48.318	0.0755
	Best	**680.630063**	680.65	684.36	680.76
	Worst	**680.632301**	681.12	901.86	681.13
G10	Mean	**7128.0197**	7844.4	8489.1	8197.3
	SD	**7.9819 × 10^1^**	349.86	358.25	395.94
	Best	**7053.124282**	7065.2	7774.5	7651.4
	Worst	**7259.656336**	8546.4	9092.6	8686.3

**Table 14:** Comparison results between MSIPKO and other algorithms using different constraint-handling strategies.

MSIPKO vs.	HMICA	BSA-SAε	SMA-GM	AGWO	IChoA
G01	+	+	−	+	+
G02	+	+	+	+	+
G03	+	+	−	**−**	**−**
G04	**=**	+	+	+	+
G05	+	+	+	+	+
G06	+	+	+	+	+
G07	+	+	+	+	+
G08	**=**	+	+	+	+
G09	+	+	+	+	+
G10	+	+	+	+	+
G11	+	+	+	+	+
G12	**=**	+	+	+	+

**Table 15:** Numerical simulation conditions and constraints on strategies of 10 comparative algorithms for solving six engineering problems.

Engineering Problems	Population Size	Iterations	FEs	Constraints on Strategies
P1: I-beam vertical deflection problem	100	500	50,000	Static penalty functions
P2: Speed reducer design problem	100	500	50,000	Static penalty functions
P3: Three-bar truss design problem	100	500	50,000	Static penalty functions
P4: Welded beam design problem	100	500	50,000	Static penalty functions
P5: Tension/compression spring design problem	100	500	50,000	Static penalty functions
P6: Pressure vessel design problem	100	500	50,000	Static penalty functions

**Table 16:** Comparison results of MSIPKO and 9 other algorithms for 6 engineering problems (1).

Functions	Statistics	MSIPKO	PKO	FLA	BKA	TTAO
P1	Mean	0.011539088	0.011539141	0.011539098	0.011669859	0.011539086
	SD	5.8307 × 10^−9^	1.3315 × 10^−7^	2.0822 × 10^−8^	4.0341 × 10^−4^	2.3164 × 10^−9^
	Best	0.011539085	0.011539085	0.011539085	0.011539085	0.011539085
	Worst	0.011539106	0.011539528	0.011539153	0.013109394	0.011539098
P2	Mean	2994.47107	2994.47113	2994.64841	3003.33858	2994.47108
	SD	5.2520 × 10^−6^	1.2865 × 10^−4^	3.5592 × 10^−1^	4.2623 × 10^0^	6.0137 × 10^−5^
	Best	2994.47107	2994.47107	2994.47236	2995.39174	2994.47107
	Worst	2994.47109	2994.47153	2995.80022	3009.72877	2994.4713
P3	Mean	263.89585	263.895895	263.896001	263.898092	263.896034
	SD	1.4485 × 10^−5^	5.8965 × 10^−5^	2.2680 × 10^−4^	7.8132 × 10^−3^	2.4729 × 10^−4^
	Best	263.895843	263.895854	263.895846	263.895844	263.895866
	Worst	263.895888	263.896061	263.896506	263.926822	263.896882
P4	Mean	1.72491801	1.72507121	1.7278333	1.72637376	1.72494056
	SD	7.1110 × 10^−5^	1.0226 × 10^−4^	6.8226 × 10^−3^	7.4049 × 10^−4^	2.1646 × 10^−4^
	Best	1.72485231	1.72488643	1.72491893	1.72549628	1.72485231
	Worst	1.72512691	1.72527729	1.74828083	1.72776065	1.72579598
P5	Mean	0.01266728	0.01267281	0.01279955	0.01272766	0.01268665
	SD	2.7694 × 10^−6^	6.2121 × 10^−6^	0.00010614	0.00011118	1.9847 × 10^−5^
	Best	0.01266523	0.01266652	0.01269552	0.01266557	0.01266527
	Worst	0.01267428	0.01268969	0.01304903	0.01312713	0.01272224
P6	Mean	6059.97668	6062.43921	6589.03996	6544.23934	6429.36137
	SD	4.1967 × 10^−1^	7.6779 × 10^0^	5.3153 × 10^2^	5.5518 × 10^2^	2.8135 × 10^2^
	Best	6059.71434	6059.74833	6059.71727	6059.71435	6077.12065
	Worst	6061.76402	6091.25867	7903.67564	7932.61173	7332.84151

**Table 17:** Comparison results of MSIPKO and 9 other algorithms for 6 engineering problems (2).

Functions	Statistics	FOX	COA	NRBO	EAO	SFOA
P1	Mean	0.013348175	0.01154031	0.011543584	0.01153909	0.01153911
	SD	1.0497 × 10^−3^	8.6685 × 10^−7^	9.3752 × 10^−6^	7.7422 × 10^−9^	1.4253 × 10^−8^
	Best	0.012028399	0.01153914	0.011539085	0.01153908	0.01153909
	Worst	0.015631203	0.01154205	0.01157396	0.01153911	0.01153914
P2	Mean	3141.042184	2994.92613	3008.606028	2994.47107	2994.47121
	SD	2.5363 × 10^2^	3.3144 × 10^−1^	8.7472 × 10^0^	2.0211 × 10^−5^	1.7063 × 10^−4^
	Best	3001.032039	2994.55934	2996.321267	2994.47107	2994.47107
	Worst	3983.427481	2995.56962	3026.688498	2994.47114	2994.47169
P3	Mean	263.8960462	263.896454	263.8958547	263.895859	263.895853
	SD	1.5251 × 10^−4^	5.2429 × 10^−4^	2.9546 × 10^−5^	3.4669 × 10^−5^	1.8380 × 10^−5^
	Best	263.8958469	263.895897	263.8958434	263.895843	263.895843
	Worst	263.8963818	263.897321	263.8959382	263.895945	263.895903
P4	Mean	2.02162212	1.72936175	1.772266732	1.72492991	1.7259699
	SD	1.8985 × 10^−1^	8.0685 × 10^−3^	4.3106 × 10^−2^	1.4279 × 10^−4^	4.1770 × 10^−4^
	Best	1.745668937	1.72504399	1.72526954	1.72485231	1.72549173
	Worst	2.427841873	1.75641958	1.85240287	1.72530084	1.72700692
P5	Mean	0.013214746	0.01281789	0.012951362	0.01266593	0.0126655
	SD	1.2683 × 10^−3^	1.5319 × 10^−4^	3.7438 × 10^−4^	2.3154 × 10^−6^	1.8155 × 10^−7^
	Best	0.012670854	0.01268791	0.012669029	0.01266523	0.01266527
	Worst	0.017733777	0.01330557	0.013934811	0.01267628	0.012666
P6	Mean	25,205.90876	6498.98538	6560.191745	6063.48687	6061.11232
	SD	3.5080 × 10^4^	5.0267 × 10^2^	5.4950 × 10^2^	1.2704 × 10^1^	1.2666 × 10^0^
	Best	6118.982612	6059.90948	6059.714335	6059.71434	6059.7767
	Worst	11,8920.0015	7903.67567	8135.496657	6120.46923	6064.47063

**Table 18:** Ten algorithms’ ranking results of 6 engineering problems by the mean value.

	MSIPKO	PKO	FLA	BKA	TTAO	FOX	COA	NRBO	EAO	SFOA
P1	3	6	4	9	1	10	7	8	2	5
P2	1	4	6	8	3	10	7	9	2	5
P3	1	5	6	10	7	8	9	3	4	2
P4	1	4	7	6	3	10	8	9	2	5
P5	3	4	7	6	5	10	8	9	2	1
P6	1	3	9	7	5	10	6	8	4	2
Total rank	10	26	39	46	24	58	45	46	16	20
Mean rank	1.6667	4.3333	6.5	7.6667	4	9.6667	7.5	7.6667	2.6667	3.3333
Final rank	1	5	6	8	4	10	7	9	2	3

**Table 19:** Six engineering problems’ solution vectors obtained by MSIPKO.

Engineering Problems	*x*_1_	*x*_2_	*x*_3_	*x*_4_	*x*_5_	*x*_6_	*x*_7_	*F*(*x*)
P1	50.0000	80	0.1707	2.8732	—	—	—	0.0115390849
P2	3.5000000	0.7000000	17	7.30000	7.715319	3.350214	5.286654	2994.471066
P3	0.788675	0.408248	—	—	—	—	—	263.895843
P4	0.2057296	3.4704886	9.0366239	0.2057296	—	—	—	1.7248523
P5	0.0516859	0.3566426	11.2933711	—	—	—	—	0.01266523
P6	0.8125	0.4375	42.0984456	176.636596	—	—	—	6059.714335

## Data Availability

The data supporting the findings of this study are available within the article.

## References

[1] Luo J., Zhou J., Jiang X. (2021). A modification of the imperialist competitive algorithm with hybrid methods for constrained optimization problems. IEEE Access.

[2] Moayed D., Gen Y. (2012). Constrained multiple-swarm particle swarm optimization within a cultural framework. IEEE Trans. Syst. Man Cybern.-Syst..

[3] Deb K. (2000). An efficient constraint handling method for genetic algorithms. Comput. Meth. Appl. Mech. Eng..

[4] Dong N., Wang Y. (2014). Novel bi-objective model-based evolutionary algorithm for constrained optimization problems. Control Theor. Appl..

[5] Bi X., Zhang L. (2016). Constrained multi-objective optimization algorithm m with adaptive ε truncation strategy. J. Electr. Inf. Technol..

[6] Zuo W., Gao Y. (2024). Solving numerical and engineering optimization problems using a dynamic dual-population differential evolution algorithm. Int. J. Mach. Learn. Cybern..

[7] Zhong C., Li G., Meng Z. (2025). Starfish optimization algorithm (SFOA): A bio-inspired metaheuristic algorithm for global optimization compared with 100 optimizers. Neural Comput. Appl..

[8] Xiao Y., Cui H., Khurma R.A., Castillo P.A. (2025). Artificial lemming algorithm: A novel bionic meta-heuristic technique for solving real-world engineering optimization problems. Artif. Intell. Rev..

[9] Alkharsan A., Ata O. (2025). Hawkfish optimization algorithm: A gender-bending approach for solving complex optimization problems. Electronics.

[10] Zhao W., Xie Y., Wang L. (2026). An effective Bezier curve-based optimization (BCO) for large-scale numerical problems and 3D unmanned aerial vehicle path planning with efficient multiple threats evasion. Adv. Eng. Inform..

[11] Xue J., Shen B. (2023). Dung beetle optimizer: A new meta-heuristic algorithm for global optimization. J. Supercomput..

[12] Yuan C., Zhao D., Heidari A.A. (2024). Polar lights optimizer: Algorithm and applications in image segmentation and feature selection. Neurocomputing.

[13] Fan S., Wang R., Kang S. (2025). A sequoia-ecology-based metaheuristic optimisation algorithm for multi-constraint engineering design and UAV path planning. Results Eng..

[14] Yu H., Jia H., Zhou J. (2022). Enhanced aquila optimizer algorithm for global optimization and constrained engineering problems. Math. Biosci. Eng..

[15] Sun B., Peng P., Tan G. (2024). A fuzzy logic constrained particle swarm optimization algorithm for industrial design problems. Appl. Soft Comput..

[16] Li G., Wang Z., Gao W. (2025). Adaptive multi/many-objective transformation for constrained optimization. IEEE Trans. Syst. Man Cybern.-Syst..

[17] Furio C., Lamberti L., Pruncu C.I. (2024). Mechanical and civil engineering optimization with a very simple hybrid grey wolf–Jaya metaheuristic optimizer. Mathematics.

[18] Meng K., Zhang J., Xu Z. (2024). Ship power system network reconfiguration based on swarm exchange particle swarm optimization algorithm. Appl. Sci..

[19] Zhao M., He Y., Tian Y. (2024). Capacity optimization of wind–solar–storage multi-power microgrid based on two-layer model and an improved snake optimization algorithm. Electronics.

[20] Boroumandfar G., Khajehzadeh A., Eslami M. (2024). A single and multiobjective robust optimization of a microgrid in distribution network considering uncertainty risk. Sci. Rep..

[21] Farhat M., Kamel S., Abdelaziz A.Y. (2025). Modified Tasmanian devil optimization for solving single and multiobjective optimal power flow in conventional and advanced power systems. Clust. Comput..

[22] Dora B.K., Bhat S., Halder S. (2024). A solution to multi-objective stochastic optimal power flow problem using mutualism and elite strategy based pelican optimization algorithm. Appl. Soft Comput..

[23] Boualem S.A.E.M., Meftah B., Debbat F. (2025). An adaptive coordinate system for constrained differential evolution. Clust. Comput..

[24] Yang H., Ren Y., Xu G. (2024). Optimization of rotary drilling rig mast structure based on multi-dimensional improved salp swarm algorithm. Appl. Sci..

[25] Abdollahzadeh B., Javadi H. (2025). The green marine waste collector routing optimization with puma selection-based neighborhood search algorithm. Clust. Comput..

[26] Wang C., Hu A., Gao Q. (2024). UAV swarm path planning approach based on integration of multi-population strategy and adaptive evolutionary optimizer. Meas. Sci. Technol..

[27] Wang X., Feng Y., Tang J. (2024). A UAV path planning method based on the framework of multi-objective jellyfish search algorithm. Sci. Rep..

[28] You G., Hu Y., Lian C., Yang Z. (2024). Mixed-strategy Harris hawk optimization algorithm for UAV path planning and engineering applications. Appl. Sci..

[29] Chen Y., Li J., Zhou L. (2024). An improved dung beetle optimizer for the twin stacker cranes’ scheduling problem. Biomimetics.

[30] Huang Z., Zhang Z., Hua C. (2024). Leveraging enhanced egret swarm optimization algorithm and artificial intelligence-driven prompt strategies for portfolio selection. Sci. Rep..

[31] Bouaouda A., Hashim F.A., Sayouti Y. (2024). Pied kingfisher optimizer: A new bio-inspired algorithm for solving numerical optimization and industrial engineering problems. Neural Comput. Appl..

[32] Ghasemi M., Golalipour K., Zare M. (2024). Flood algorithm (FLA): An efficient inspired meta-heuristic for engineering optimization. J. Supercomput..

[33] Wang J., Wang W., Hu X. (2024). Black-winged kite algorithm: A nature-inspired meta-heuristic for solving benchmark functions and engineering problems. Artif. Intell. Rev..

[34] Zhao S., Zhang T. (2024). Triangulation topology aggregation optimizer: A novel mathematics-based meta-heuristic algorithm for continuous optimization and engineering applications. Expert Syst. Appl..

[35] Mohammed H., Rashid T. (2023). FOX: A FOX-inspired optimization algorithm. Appl. Intell..

[36] Jia H., Rao H., Mirjalili S. (2023). Crayfish optimization algorithm. Artif. Intell. Rev..

[37] Sowmya R., Premkumar M. (2024). Newton–Raphson-based optimizer: A new population-based metaheuristic algorithm for continuous optimization problems. Eng. Appl. Artif. Intell..

[38] Rodan A., Al-Tamimi A.K., Al-Alnemer L., Mirjalili S., Tiňo P. (2025). Enzyme action optimizer: A novel bio-inspired optimization algorithm. J. Supercomput..

[39] Zhang C., Lin Q., Gao L. (2015). Backtracking search algorithm with three constraint handling methods for constrained optimization problems. Expert Syst. Appl..

[40] Thakur G., Pal A., Mittal N. (2024). Slime mould algorithm based on a Gaussian mutation for solving constrained optimization problems. Mathematics.

[41] Ma C., Huang H., Fan Q., Wei J., Du Y., Gao W. (2022). Grey wolf optimizer based on Aquila exploration method. Expert Syst. Appl..

[42] Preeti, Kaur R., Singh D. (2022). Dimension learning-based chimp optimizer for energy efficient wireless sensor networks. Sci. Rep..

